# Modelling and simulation of species transport in capillary-fed electrolysis cells

**DOI:** 10.1039/d6fd00128a

**Published:** 2026-10-27

**Authors:** Marco De Paoli, Jeffery A. Wood, Albertus W. Vreman, Dominik Krug

**Affiliations:** a Institute of Fluid Mechanics and Heat Transfer, TU Wien Getreidemarkt 9 Wien 1060 Austria; b Soft Matter, Fluidics and Interfaces, MESA+ Institute for Nanotechnology, J. M. Burgers Centre for Fluid Dynamics, University of Twente Enschede 7500 AE Netherlands; c Nobian Innovation Center Hengelosestraat 500 7521 AN Enschede The Netherlands; d Power and Flow Group, Department of Mechanical Engineering, Eindhoven University of Technology PO Box 513 Eindhoven 5600 MB The Netherlands; e Chair of Fluid Mechanics and Institute of Aerodynamics, RWTH Aachen University Aachen 52062 Germany; f Physics of Fluids Group, Max Planck Center for Complex Fluid Dynamics, J. M. Burgers Centre for Fluid Dynamics, University of Twente Enschede 7500 AE Netherlands d.krug@aia.rwth-aachen.de

## Abstract

In so-called capillary-fed electrolysis cells (Hodges *et al.*, *Nature Commun.*, 2022, **13**, 1304), electrolyte is resupplied through capillary forces in a porous structure contained between the two electrodes. This configuration enables largely bubble-free operation, resulting in low cell potentials even at high current densities, and this approach is therefore highly promising for terrestrial as well as space applications. While sustained operation has been demonstrated experimentally, the mechanism enabling this remains unclear, especially given the expected rise in electrolyte concentration over time and the risk of salt precipitation in the porous separator. In this study, our goal is to identify possible mechanisms that could act to limit the maximum electrolyte concentration in the separator. To this end, we present a one-dimensional model for the electrolyte transport in the porous separator, based on which we evaluate the limits of capillary feeding as a function of electrode size, properties of the porous structure, and current density. Our estimates suggest that any potential diffusio-osmotic effect remains negligible in the present setup, and no stable operation seems possible within solubility limits at meaningful electrode sizes and current densities within the 1D model. Three-dimensional simulations of fluid flow and mass transport for a 1 cm^2^ electrode on a larger separator, performed using COMSOL Multiphysics, demonstrate that allowing diffusion in all spatial directions significantly enlarges the stable operating window, while buoyancy has only a very limited effect in the present configuration. Simulations accounting for solutal Marangoni effects at the separator surface indicate that the resulting Marangoni stresses are sufficiently strong to induce a recirculating flow within the separator, thereby lowering the maximum electrolyte concentration in the electrode region. This appeared to allow continuous operation without exceeding solubility limits for the maximum simulation time of 5 hours at 0.4 A cm^−2^, similar to what was achieved experimentally.

## Abstract 2

Multidirectional diffusion and solutal Marangoni flow mitigate electrolyte accumulation in capillary-fed electrolysis cells, providing a possible mechanism for sustained operation without salt precipitation.

## Graphical abstract

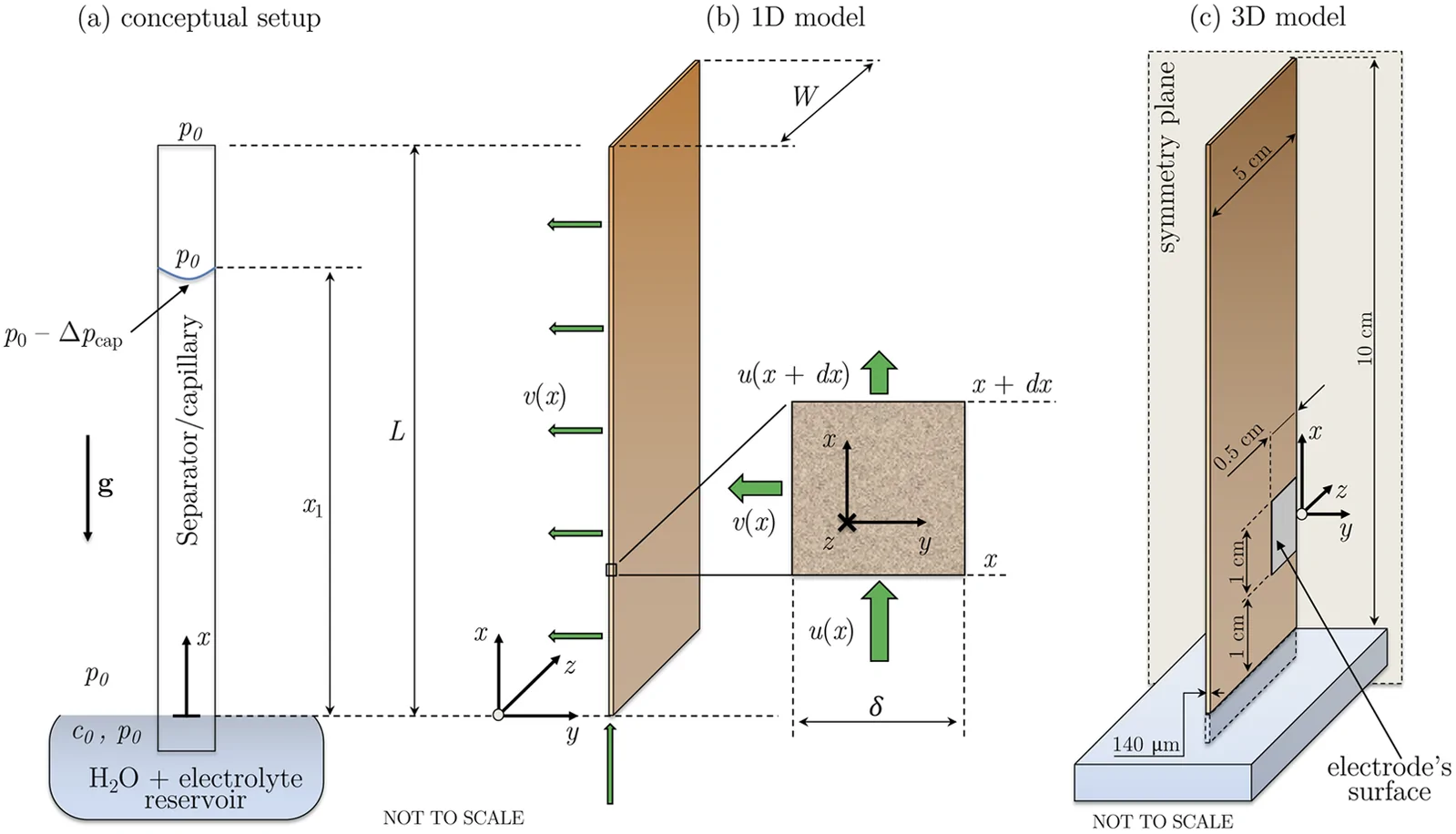

## Introduction

Despite their fascinating physics and importance in numerous applications,^1^ gas bubbles are generally undesirable in electrolyzers. They nucleate from supersaturated dissolved gas at the electrode surface, where they obstruct active reaction sites, hinder the transport of reactants and products, and increase the ohmic resistance of the electrolyte, thereby reducing the overall efficiency of the electrochemical process.^2^ The associated efficiency losses are considered a major barrier to the scale-up of electrolyzers to the high current densities required for economically viable terrestrial green hydrogen production.^3^ It is also highly relevant for space applications, where water electrolysis is used for oxygen generation, *e.g.*, on the International Space Station. Due to the near absence of buoyancy-driven flows under microgravity conditions, efficient bubble removal becomes even more challenging in space-based electrolyzer systems.^4^

Besides active and passive strategies^3^ for mitigating bubble-related losses, such as electrode structuring^5,6^ and exploiting coalescence-induced bubble departure,^7^ bubble-free concepts seem particularly attractive to mitigate bubble effects during electrolysis. By severely limiting or potentially preventing bubble formation altogether, they avoid bubble-induced efficiency losses.^3,8^ This has been demonstrated to improve cell performance compared with a conventional zero-gap design by ref. 8. These authors also argue that bubble-free operation may also simplify the overall system design and reduce the associated energy consumption by eliminating the need for auxiliary units such as gas–liquid separators and water chillers.

Related studies to date have employed gas-diffusion electrodes consisting of thin catalyst layers deposited on porous hydrophobic substrates, such as expanded PTFE (ePTFE) membranes^9–11^ or nanoporous polyethylene.^12^ At low current densities, these systems have demonstrated bubble-free operation together with substantially improved performance. A particularly interesting concept in this context was introduced by Hodges *et al.* (2022),^8^ who gently resupplied the electrolyte through capillary feeding in a porous separator in between the electrodes. For a small test cell, they reported an energy efficiency of 98% at 0.5 A cm^−2^ and 80 °C, and continuous operation at room temperature for up to 30 days at 0.4 A cm^−2^.

This approach is therefore highly attractive from a practical perspective, but it also raises several intriguing scientific questions. One concerns the limits of capillary-driven transport: how do the maximum achievable electrode size and current density depend on the properties of the separator?

A second question relates to the mechanism that prevents the electrolyte concentration within the separator from increasing beyond its solubility limit during continuous operation. Since only water (the solvent) is consumed during electrolysis, one would expect a gradual accumulation of dissolved salt and, eventually, crystallization within the separator between the electrodes. To our knowledge, the mechanism preventing such concentration buildup remains elusive.

Addressing these questions may provide fundamental insights that enable further optimization and scaling of this design concept, as well as its transfer to other applications, including electrolysis systems for space exploration. In this work, we investigate this problem using a combined analytical and numerical approach. A one-dimensional model of capillary-driven flow, diffusion and viscous drag is used to establish upper bounds on electrode size and current density for solubility-limited operation. Since this approach cannot capture lateral transport or localized concentration buildup near a finite electrode, we complement it with three-dimensional simulations that additionally account for buoyancy and solutal Marangoni effects. Together, these models allow us to test which mechanisms, if any, can explain the sustained operation observed experimentally without exceeding the KOH solubility limit.

## Methods

### Problem formulation

We consider a capillary-fed separator configuration similar to that employed by Hodges *et al.*^8^ The setup consists of a reservoir containing an aqueous KOH solution into which a vertically oriented porous separator is immersed. During operation, capillary forces within the separator continuously replenish the water consumed by the electrolytic reactions occurring at the electrodes located on either side of the separator. A sketch of the flow configuration is provided in Fig. 1(a). In the following, we will focus on flow and transport phenomena within the separator by means of an analytical model and simulations of the system.

**Fig. 1:**
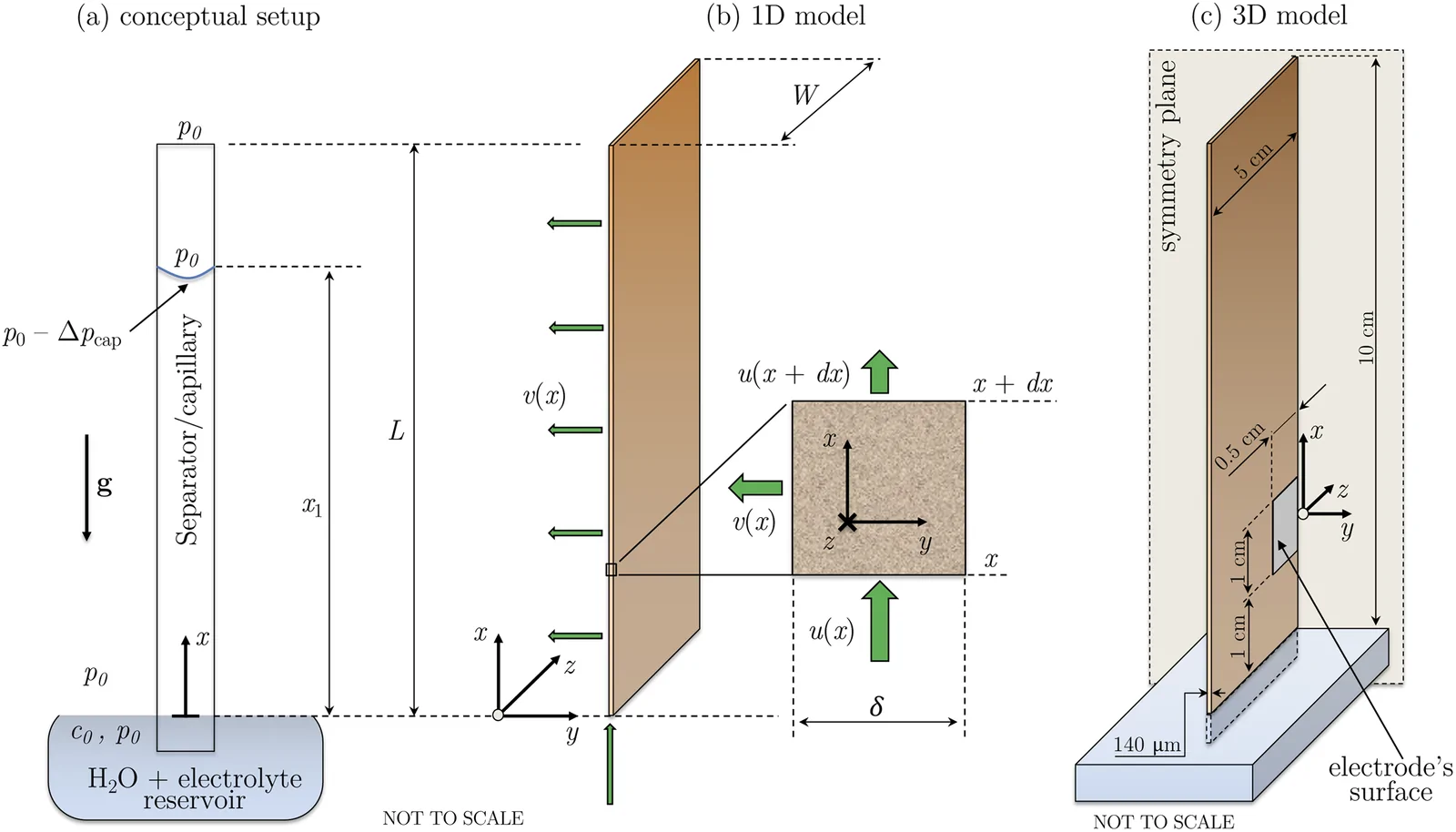
Sketch of the flow configuration for the capillary-fed separator. (a) The electrolyte reservoir (aqueous solution of KOH, considered at constant concentration (*c*_0_) feeds the vertically aligned separator (gravity acceleration *g* is also indicated). The capillary pressure drop Δ*p*_cap_ is considered to be localized at the top boundary, where the liquid–air interface is located, at distance *x*_1_ from the reservoir. (b) Three-dimensional visualization of the separator with indication of the membrane dimensions (*δ*, *L*, *W*). A 2D representation of a chunk of membrane is also reported, where the superficial flow velocities in streamwise (*x*) and wall normal directions (*y*), *u* and *v*, respectively, are also indicated. (c) Geometry and setup used in the 3D numerical simulations, with explicit indication of the symmetry boundary condition and the geometrical details (dimension of separator, electrode and relative position).

The reservoir conditions, indicated in Fig. 1(a), are assumed to be constant during the process, namely a solute (in this case, KOH) concentration equal to *c*_0_ = 6 mol l^−1^, corresponding to 27 wt% KOH. The pressure at the bottom of the separator matches the ambient pressure (*p*_0_), which is also constant. The separator is characterized by a porosity *ε*, pore size *r* and contact angle *θ*. Capillary forces due to the liquid–air interface (located within the separator at distance *x*_1_ from the reservoir) are considered to be the main driving forces of the flow: a pressure difference Δ*p*_cap_ = 2*γ* cos *θ*/*r* arises due to capillary effects induced by the presence of the narrow channels within the medium. Here, *γ* is the surface tension of the liquid.

### 1D model

To develop the analytical model, a one-dimensional approximation of the problem is considered, as illustrated in Fig. 1(b): we assume the flow to be uniform in the spanwise (*z*-) direction (∂·/∂*z* = 0), and we only consider *y*-averaged (*i.e.*, across the thickness) flow quantities, such that the flow and scalar fields (namely, flow velocity components *u* and *v*, solute concentration *c*, and pressure *p*) vary only along the vertical direction *x*.

To account for the consumption of water during electrolysis, we consider the molar flux of hydrogen, *J*_H_2__ [mol_H_2__ m^−2^ s^−1^], which is related to the current density *i* [A m^−2^] *via*1
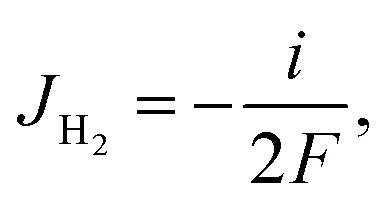
where *F* = 9.648 × 10^4^ C mol^−1^ is the Faraday constant. Since every water molecule gives one molecule of H_2_, the molar fluxes of H_2_ and H_2_O are equivalent, and therefore the effective (*i.e.*, accounting for both sides) superficial velocity *v*(*x*) of the liquid in the lateral direction can be computed as2
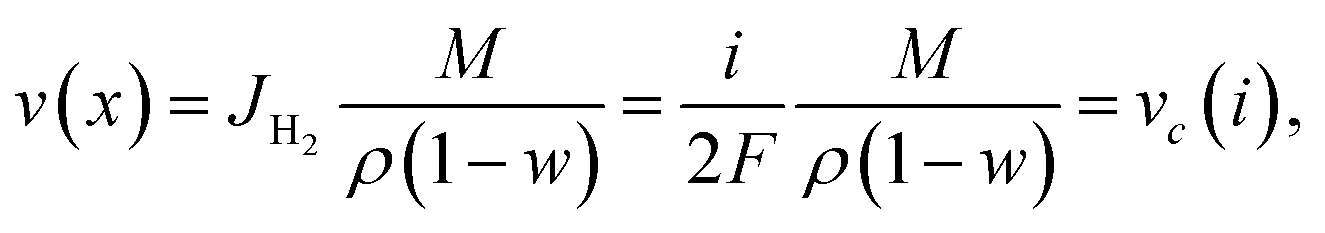
where *M* is the molar mass of H_2_O and *ρ*(1 − *w*) is the density of H_2_O in the solution, where *ρ* is the density of the solution and *w* is the weight fraction of KOH. Finally, we observe that, assuming a uniform temperature and current density along the *x*-direction, the superficial velocity is also uniform and equal to *v*(*x*) = *v*_*c*_(*T*, *i*). In the following, we will apply this approximation such that *v*(*x*) = *v*_*c*_.

### 3D model

To provide more detailed insights, transient 3D simulations were carried out in COMSOL Multiphysics 6.3, solving for the transport of species and fluid-velocity components based on concentration-dependent physical properties with the porous separator treated in a Darcian fashion and, for simplicity, a concentration-dependent diffusivity for KOH/water (a Fickian-type approach) was used in order to determine the concentration (weight fraction) of KOH (and water) and the velocity/pressure profile. The physical properties (density, viscosity, surface tension and diffusivity) of the system were all taken as functions of KOH concentration, as described in the SI. The system geometry employed in the simulations is as shown in Fig. 1(c). Note that, unlike the configuration used for the 1D model, the electrode here is limited to a patch of 1 cm × 1 cm placed 1 cm above the electrolyte in a much larger separator layer mimicking the setup used in ref. 8.

The electrode is placed on a 10 cm × 140 µm × 10 cm porous separator (pore radius = 8 µm and porosity of 0.8) immersed in a much larger water reservoir, with the fluid temperature at 80 °C and a reservoir concentration of 27 wt% KOH. The system was solved using a symmetry plane in the third dimension (*z*-direction) dividing the separator in half in all cases.

The density at the reservoir inlet (27 wt% KOH) was ∼1226 kg m^−3^ and the viscosity was 0.8431 mPa s (using the pure water correlation of Likhachev (2003)^13^ as part of the calculation), as described by Hodges *et al.* (2023),^14^ and varied according to the KOH weight fraction. In the simulations, all physical properties were concentration- and temperature-dependent, although, in our simulations, we consider an isothermal operating case at 80 °C, with gravity included. The impact of other system components, such as the gas-diffusion electrodes, *etc.*, were neglected – the electrode reactions were assumed to take place on a 2D plane.

In the 1D model, we effectively assume a lumped net reaction along the height, resulting in a net outflow velocity *v*_*c*_. The impact of this assumption was tested by considering the physical case of asymmetric reactions in the 3D case (consumption and generation of H_2_O at the cathode and anode, respectively).^15^ In this asymmetric case, the cathode was treated as an outflow/outflux, and the anode as an inflow/influx, with the magnitudes varying according to the individual electrode-reaction stoichiometry.

We also explore the possible role of solutal Marangoni convection in this system. To this end, we assumed that all vertical surfaces of the separator were wetted and imposed the tangential stress resulting from spatially varying solute concentrations at those boundaries (neglecting the possibility of Marangoni convection at the upper boundary of the separator after initial simulation results were available that indicated that this effect was negligible there).

The equations and boundary conditions are described in the SI in more detail. Numerically, the simulations were carried out using a structured rectangular prism mesh with a P1–P1 formulation for velocity and pressure and first-order Lagrange elements for concentration (weight fraction of KOH). Mesh independence was visually assessed through successive refinement until changes within the velocity profile appeared negligible.

## Results and discussion

### 1D model

#### Volume (or mass) conservation

Employing the one-dimensional approximation, one can write a volume conservation equation for the fluid phase. At a distance *x* from the reservoir, the volume flow rate *Q* [m^3^ s^−1^] is

3 *Q*(*x*) = *u*(*x*)*Wδ*,

where *u*(*x*) is the vertical superficial fluid velocity averaged over the membrane thickness *δ*, and *W* denotes the width of the separator. From volume conservation it then follows that4
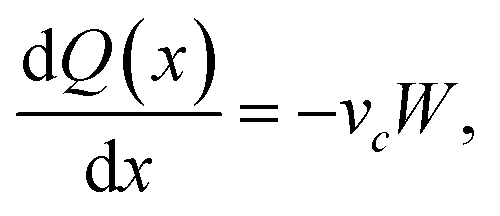
which can be integrated to yield

5 *Q*(*x*) = *v*_*c*_*W*(*x*_1_ − *x*),

where *x*_1_ represents the maximum transport distance that can be sustained by capillary driving. In the following, *x*_1_ is determined as a function of the relevant flow parameters. Note that, in the present setup, the electrode height also scales with *x*_1_, and hence so does the total current. From eqn (5), it further follows that the maximum flow rate is obtained at the bottom (*x* = 0) and corresponds to *Q*_max_ = *v*_*c*_*Wx*_1_.

#### Steady-state concentration profile

The linear variation of *Q*(*x*) according to eqn (5) also implies a linear variation of the gap-averaged flow velocity, *u*(*x*) = *Q*(*x*)/(*Wδ*). On this basis, we can investigate the steady-state solution for the variation *c*(*x*) of the electrolyte concentration along the separator.

Assuming steady-state conditions (d*·*/d*t* = 0), the Fickian transport equations reduce to a balance between convective and diffusive fluxes according to6
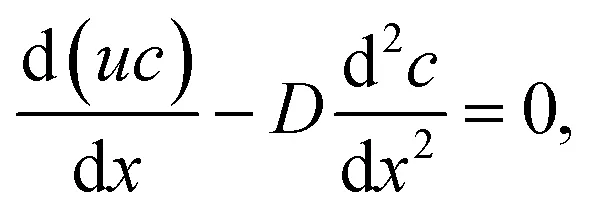
where *D* is the diffusion coefficient of KOH. Note that the effect of porosity is neglected here. In a porous medium, diffusion is reduced due to the combined effects of porosity and tortuosity, for example, according to *D*_eff_ = *Dε*^3/2^ when adopting the Bruggeman relation.^16,17^ Consequently, the results presented in this section are independent of the porous microstructure and should therefore be regarded as an optimistic upper bound on the actual operating limits.††All the results shown for the 1D model are for 27 wt% KOH at temperature *T* = 80 °C (unless mentioned otherwise), *i.e.*, these values are used to compute density (*ρ* = 1226.0 kg m^−3^), dynamic viscosity (*µ* = 0.84310 mPa s) and surface tension (*γ* = 0.0792 N m^−1^) as described by Hodges *et al*. (2023).^14^ For the contact angle, we assume *θ* = 70.3° (ref. 8) and for the diffusion coefficient *D* = 8 × 10^−9^ m^2^ s^−1^; see Bhatia *et al*. (1968).^18^ The membrane thickness is set to *δ* = 140 µm.^8^ Inserting the linear profile of *u*(*x*), which follows from eqn (3) and (5), this can be rewritten as:7
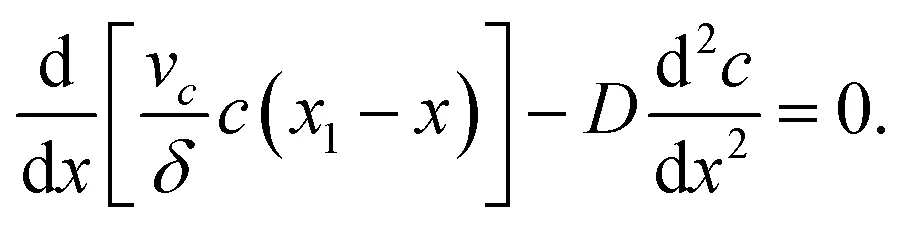


Introducing the dimensionless variables c* = *c*/*c*_0_ and x* = *x*/*x*_1_, and the Péclet number8
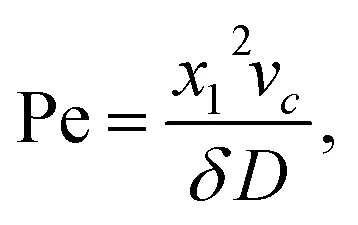
the dimensionless form of eqn (7) is obtained:9
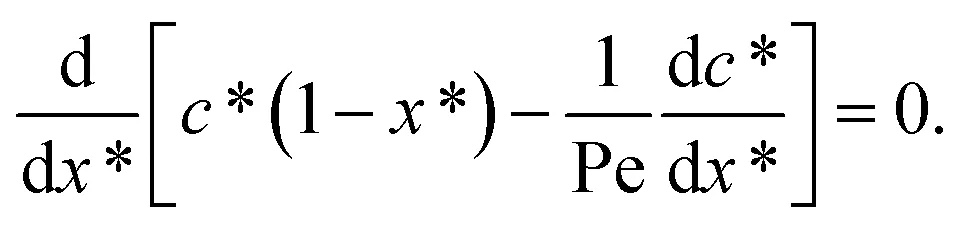


Eqn (9) implies that the net flux in parentheses (*uc* − *D*d*c*/d*x* in dimensional form) is constant along the height. Applying the no-flux boundary condition at the top, 
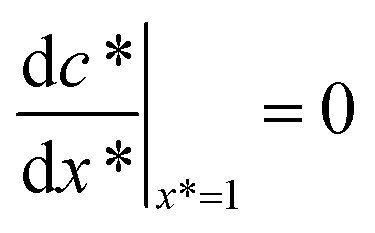
, then shows that this constant must vanish, yielding10
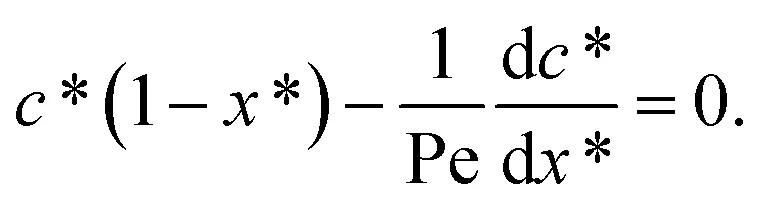
This can be solved for the non-dimensional concentration profile c*(x*) using a fixed concentration at the bottom, c*(x* = 0) = 1, which results in11
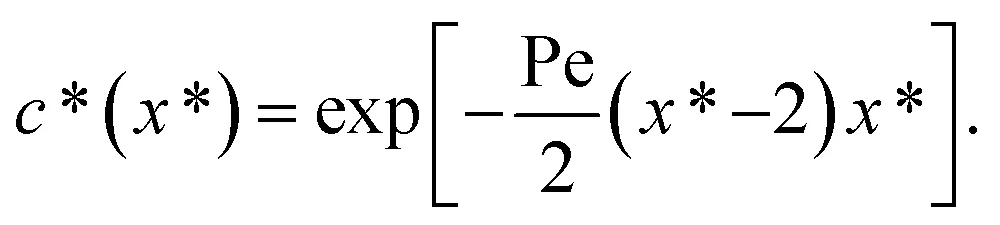
Examples of concentration distributions predicted by eqn (11) are reported in Fig. 2(a) for different value of Pe. Due to the dependence on Pe, the concentration values increase exponentially with increasing electrode height or current density (*i.e.*, increasing *v*_*c*_).^18^ Practically, however, the concentration cannot grow indefinitely, and the maximum value of concentration that can be achieved is set by the maximum solubility, that is *c*_max_. Adopting *c*_max_ ≈ 15 M and *c*_0_ ≈ 6 M,^19^ it follows that 
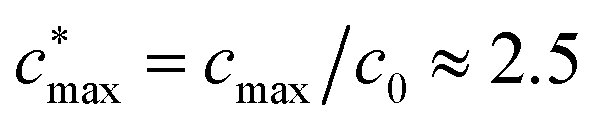
. To obtain this value at the top (x* = 1), the corresponding Péclet number obtained from eqn (11) is12
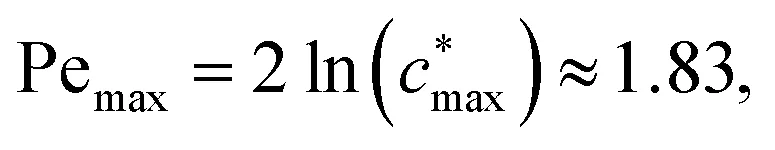
and the corresponding profile is indicated in red in Fig. 2(a). Solutions of the 1D diffusion model at larger Pe values are not practically realizable. To place this result into the context of the practical problem, the region of the parameter space (*i*, *x*_1_) for which realizable solutions exist (Pe ≤ Pe_max_) is highlighted in blue in Fig. 2(b).

**Fig. 2:**
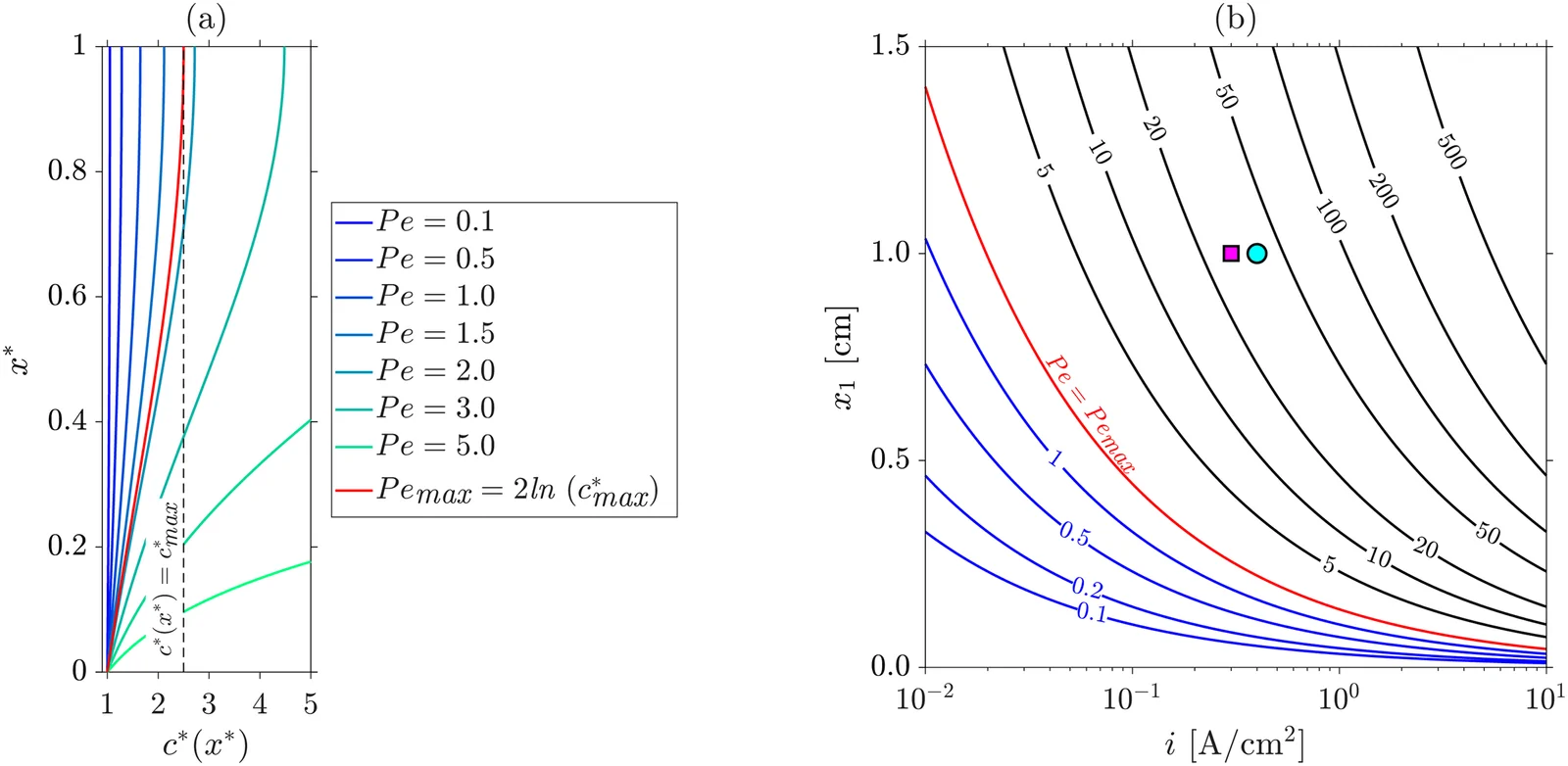
(a) Concentration distribution according to eqn (11) for different values of Pe. The red line corresponds to the Péclet number for which the solubility limit 
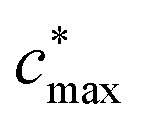
 is first reached at the top; see eqn (12). (b) Péclet number as a function of current density *i* and distance travelled *x*_1_. Contours relative to Pe < Pe_max_, Pe = Pe_max_ and Pe > Pe_max_ are shown in blue, red and black, respectively. The experimental data relative to the work of Hodges *et al.*^8^ are also reported (symbols). These data correspond to an electrode size *x*_1_ = 1 cm and to a current density of *i* = 0.3 A cm^−2^ (square) and *i* = 0.4 A cm^−2^ (circle), corresponding to *T* = 80 °C and room temperature (*T* = 20 °C), respectively.

The maximum attainable electrode height does not even reach 1.5 cm at current densities as low as 10^−2^ A cm^−2^ and decreases to approximately 0.1 cm at 1 A m^−2^. We now consider in this context the experiments of Hodges *et al.*,^8^ which we report as symbols in Fig. 2(b). These experimental data correspond to an electrode size *x*_1_ = 1 cm and to a current density of *i* = 0.3 A cm^−2^ (square) and *i* = 0.4 A cm^−2^ (circle), corresponding to *T* = 80 °C and room temperature (*T* = 20 °C), respectively. Since these points fall entirely within the region Pe > Pe_max_, it is evident that the simple 1D diffusion model cannot explain the sustained operation observed experimentally by Hodges *et al.*^8^ Note that the increase in the effective diffusion coefficient at higher concentrations and due to non-ideality-related thermodynamic effects^20^ is neglected here. While this may slightly affect the quantitative results, it is not expected to alter the qualitative conclusions, as the values of *D* remain of the same order of magnitude. This is also confirmed by the results of our 3D simulations, where this effect is accounted for. The maximum diffusivity closer to solubility limits would be ∼9 × 10^−9^ m^2^ s^−1^, which is only approximately a 12.5% difference.

These findings suggest that additional mechanisms, not captured by the present model, may play an important role in this problem. In this context, Hodges *et al.*^8^ alluded to osmotic effects arising from concentration gradients. In the present configuration, so-called diffusio-osmosis may arise due to interactions between the solute gradient with the liquid/solid interface at the pore walls and can be thought of as a solid-fluid analogue to the Marangoni effect.^21–23^ We can estimate the degree of diffusio-osmotic transport *via* a gradient in KOH to assess the potential impact of osmotic-related flows on the operation of a capillary electrolyzer. Diffusio-osmosis is typically studied at low (dilute) concentrations and even studies involving more concentrated solutions involving diffusio-osmosis or the related phoretic effects are performed well below 6 M,^24^ as higher salt concentrations would be expected to reduce the degree of diffusio-osmotic flow. However, despite this, we can make some estimations as to the maximum possible diffusio-osmotic slip velocity we would expect.

The expression for diffusio-osmotic slip velocity of a 1 : 1 electrolyte is given as:^22^13
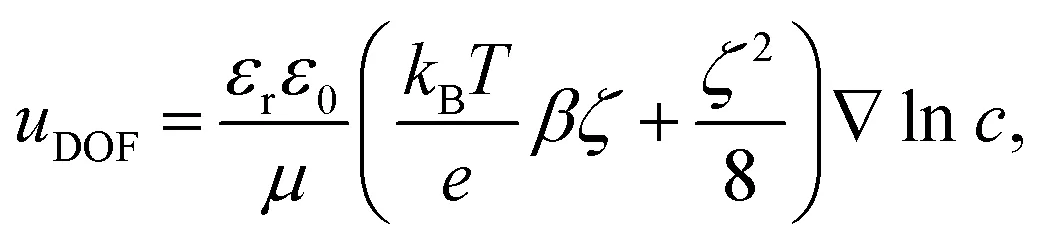
where in eqn (13)*ε*_r_ is the dielectric constant (relative permittivity) of the solvent, *ε*_0_ is the vacuum permittivity (8.85 × 10^−12^ F m^−1^), *µ* is the solvent viscosity, *k*_B_ is Boltzmann’s constant (1.38 × 10^−23^ J K^−1^), *T* is the temperature in K, *β* = (*D*_+_ − *D*_−_)/(*D*_+_ + *D*_−_) a term related to the relative speed of cation and anion, *ζ* is the zeta potential of the wall and ∇ ln *c* is the gradient of the logarithm of the solute (KOH in this case).^22^

At high concentrations beyond 6 M, specific values of zeta potential are unavailable as traditional electrokinetic measurement techniques are typically limited to far lower concentrations (0.1 M) and have been extrapolated using theories derived for dilute salt electrostatics to moderately higher concentrations.^25^ For this reason, we take zeta-potential values based on lower pH and ionic strengths for polyether sulfone (PES) ultrafiltration membranes, as the work of Hodges *et al.* (2022)^8^ used a PES separator. These values should be overestimates, in order to try to provide an upper limit on how large of a diffusio-osmotic slip would seem to be possible based on a linear concentration gradient ranging from 6 M to 15 M over a 1 cm distance.

In order to calculate the diffusio-osmotic slip flow (DOF), a number of physical parameters are required. *β*, related to the relative difference in the diffusion coefficients of the cation and anion, was taken as −0.456 from the values at infinite dilution at 298 K (ref. 26) and it was assumed that this ratio is effectively constant *vs.* temperature. The dielectric constant was taken as that of pure water at 80 °C (∼60),^27^ meaning it overestimates the DOF as higher ionic strengths will also reduce this value. The viscosity (*µ*) was taken as that of 27 wt% KOH at 80 °C (∼60),^14^ which would be the smallest value found over the height and therefore also favour overestimation of the overall impact of diffusio-osmosis. Zeta potentials from −15 to −40 mV were used; these values are consistent with measurements for polyethersulfone membranes at much lower pH values (≤10), much lower ionic strengths, and at room temperature.^28,29^ Using values in this range likely vastly overestimates the value of DOF that would be expected.

Based on these values, the maximum diffusio-osmotic slip velocity (which would occur along the *z*-axis) determined from eqn (13) ranged from 20 nm s^−1^ to 70 nm s^−1^. These values occur closest to the inlet and decrease in magnitude along the height of the system, so they already represent the maximum possible contribution. In comparison, the convective velocity at 0.1 A cm^−2^ in the normal direction would be expected to be approximately 20 to 50 times larger than even this maximum DOF velocity. Even if the zeta potential became much larger in magnitude (ex. ∼−70 mV), the diffusio-osmotic contribution would be ∼10× smaller than the convective transport at the lowest current density.

To reiterate, this estimate of diffusio-osmotic slip is already a considerable overestimate due to: (i) the screening of wall charges expected at high ionic strengths (smaller-magnitude zeta potential), and (ii) the lower dielectric constant from high ionic strength that would occur under these conditions and which would vary considerably more over the height due to viscosity increases with increasing KOH concentration. Even if none of these effects occurred, the estimated value is still considerably lower than the convective influx velocity in the same direction. This would seem to indicate that diffusio-osmotic-related recirculation cannot by itself provide an explanation for the experimental observations of continuous operation of a capillary electrolyzer at the lowest current density we explored.

We will revisit this point using results from three-dimensional simulations to identify mechanisms that may explain the sustained operation reported in the literature. Before doing so, however, we first examine the limits of capillary-driven transport, which have not been considered thus far, using the one-dimensional model.

#### Pressure drop

It remains to be evaluated whether the flow implied by the linear variation of *Q*(*x*) according to eqn (5) can be sustained by the available driving forces. Concretely, the feasibility condition is that the net pressure drop associated with the imposed flow distribution does not exceed the maximum capillary pressure. To assess this, it is necessary to determine how the pressure drop along the separator depends on *u*(*x*).

The flow through the porous separator leads to a hydraulic loss due to the viscous drag. Assuming that the separator is a uniform medium and that the flow is steady and unidirectional, the mean velocity *u*(*x*) is proportional to the pressure gradient in the flow direction 
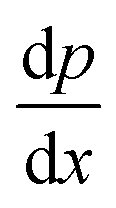
 following Darcy’s law:^30^14
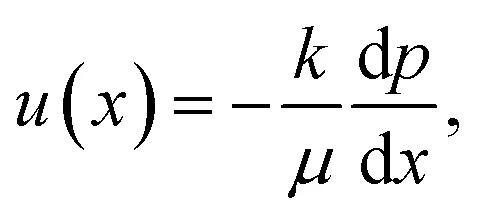
where *k* and *µ* are the medium permeability and the dynamic fluid viscosity, respectively. To account for the presence of buoyancy forces directed in the flow direction, the momentum (Darcy) equation (eqn (14)) is rewritten as15
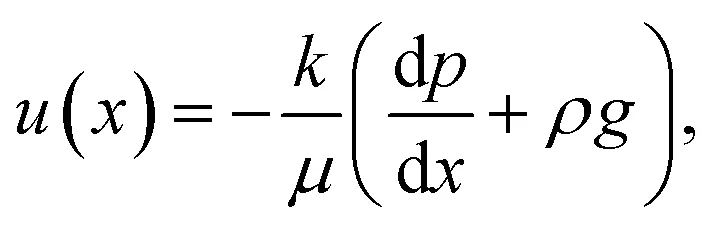
where the gravity force (acceleration *g*) is considered and leads to a hydrostatic pressure gradient of 
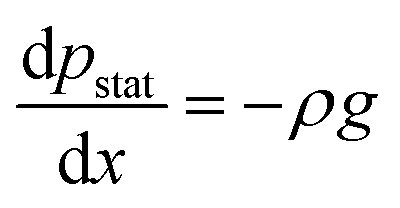
 along the separator. As a result, 
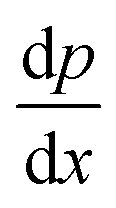
 and 
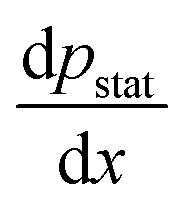
 are combined into the Boussinesq–Darcy equation (eqn (16)) as an effective pressure gradient. The flow rate that can be sustained according to eqn (3) and eqn (15) is therefore16
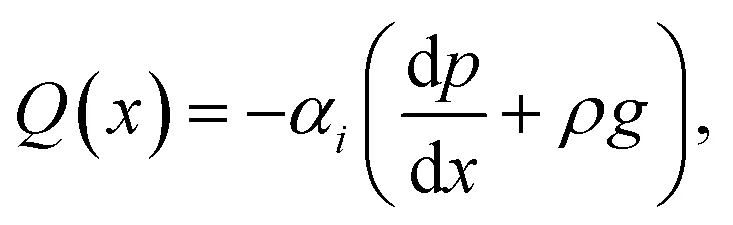
where *α*_*i*_ is an effective friction coefficient of the porous material, for which two different derivations based on a beads pack (*α*_1_) and a network of capillary channels (*α*_2_, used in this 1D model and also in ref. 8) are provided in the SI, both leading to the same functional form.

#### Limits of capillary feeding – model

In order to explore the feasibility limits of capillary feeding, we must integrate eqn (16) to yield the total pressure difference along the separator. Using eqn (5), we rewrite eqn (16) as17
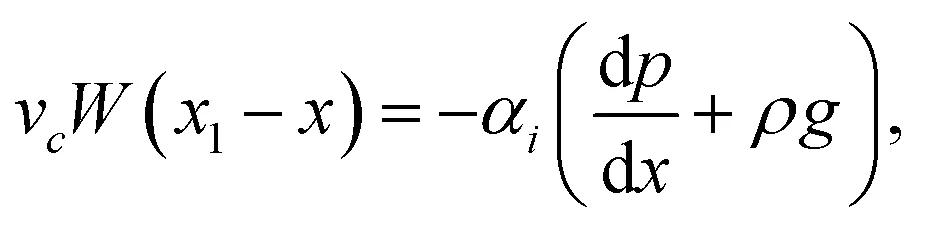
which is rearranged and integrated between *x* = 0 and *x* = *x*_1_^−^. Note that here we choose *x*_1_^−^ and not *x*_1_ to be able to include the capillary pressure jump in the pressure boundary at the top interface, which is given by *p*(*x*^−^_1_) = *p*_0_ − Δ*p*_cap_. This represents the limit of feasibility, in which the resulting pressure drop exactly equals the capillary pressure. We obtain18
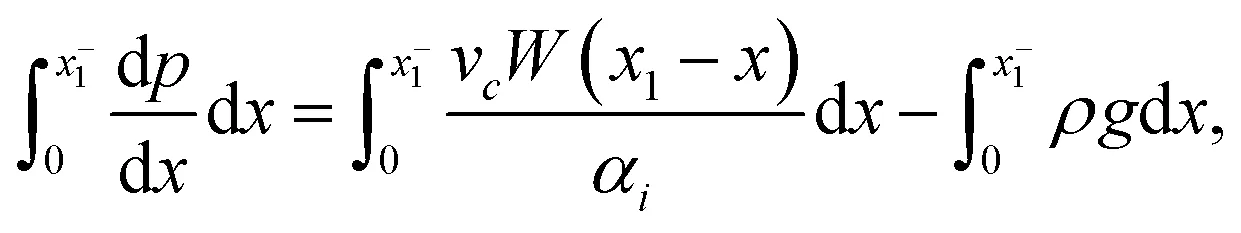
19
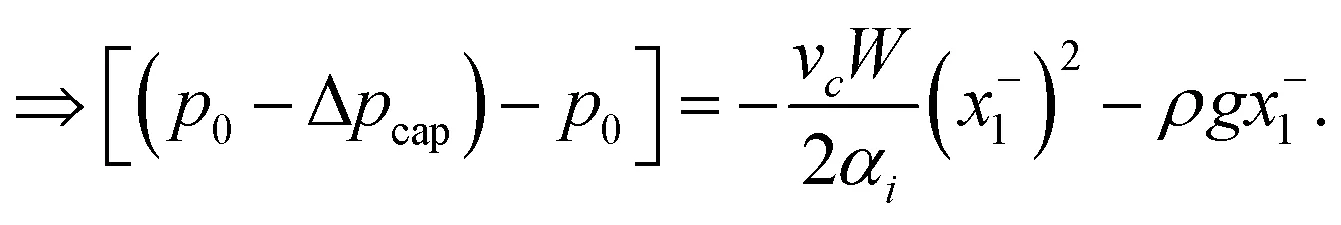
Inserting 
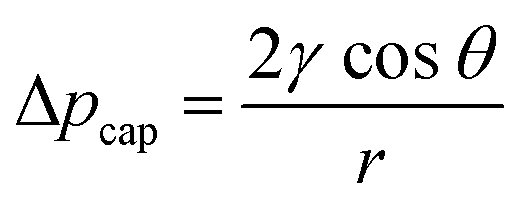
 results in a balance between the capillary pressure driving the flow and the opposing forces due to viscous drag and gravity given by20
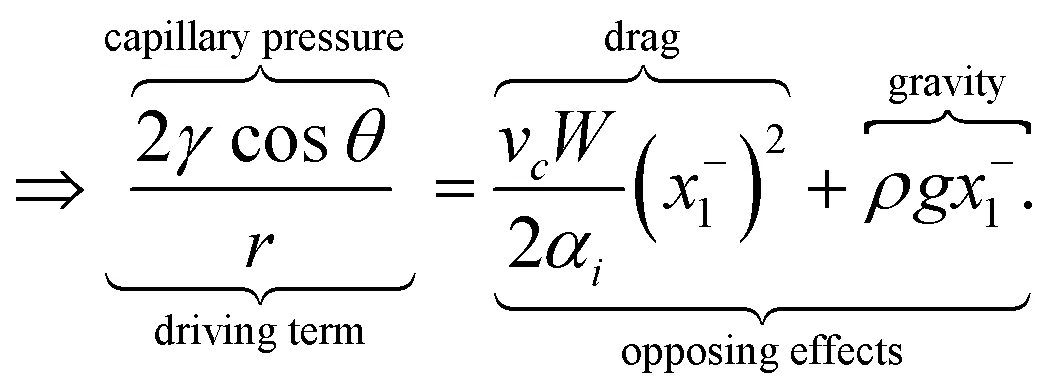
When solving for the maximum achievable height *x*_1_ in the following, we also consider limiting cases in which the capillary pressure is balanced by individual terms on the right-hand side of eqn (20), in order to gain further insight into the relevant mechanisms in specific parameter regimes.

#### Capillary pressure + gravity

As a first reference, we consider the limit of vanishing flow (*u* → 0) in which the capillary pressure is balanced by gravity only. For this case, we recover from eqn (20) the classical expression for the capillary rise:21
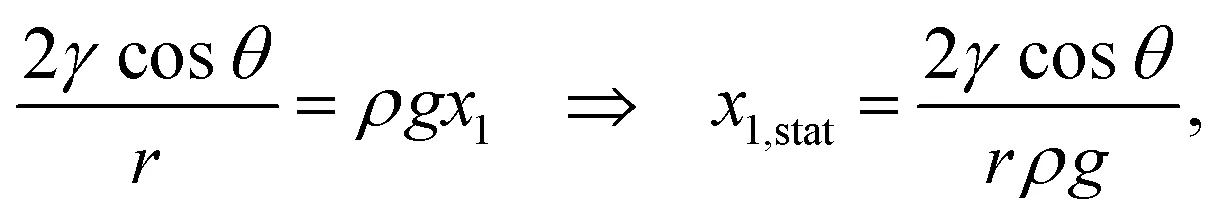
where *x*_1,stat_ represents the upper bound for *x*_1_ in the case of capillary-driven flow.

#### Capillary pressure + drag

Next, we consider the case in which the only driving source is the capillary pressure, and the opposing force is represented exclusively by the friction (dissipative term of the Darcy law). As a result, eqn (20) reduces to:22
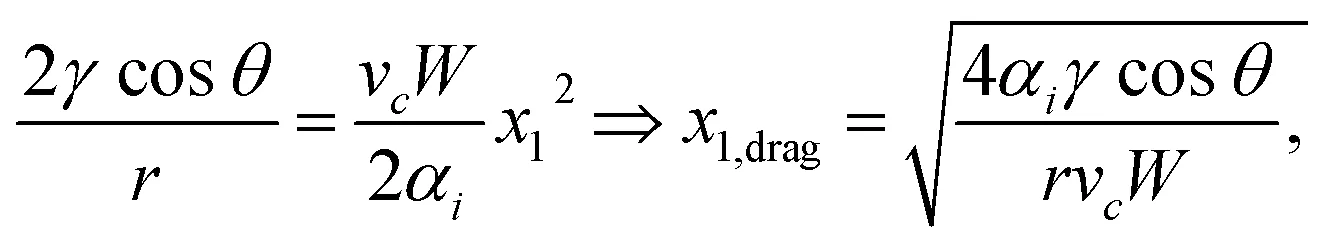
with *x*_1,drag_ denoting the corresponding maximum height.

#### Capillary pressure + gravity + drag

In the presence of both opposing mechanisms in eqn (20), the resulting solution is given by23
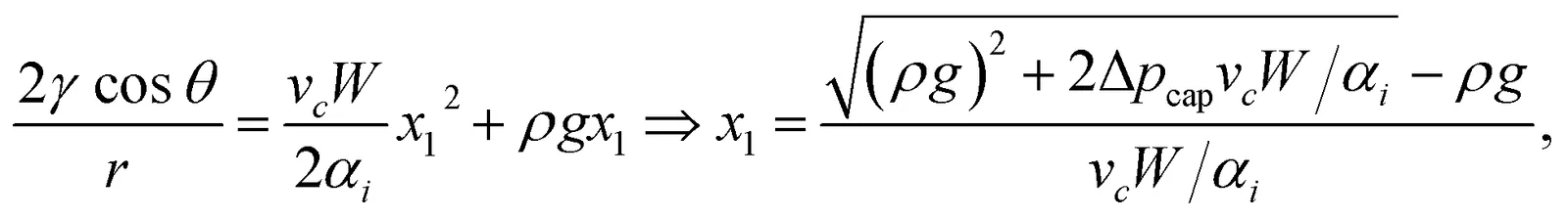
where the resulting value of *x*_1_ is lower than both *x*_1,stat_ and *x*_1,drag_.

#### Limits of capillary feeding – discussion

To provide an overview of the general behaviour predicted by the model, Fig. 3(a) shows *x*_1_ as a function of the pore radius *r*, while keeping the current density and porosity fixed at *i* = 1.00 A cm^−2^ and *ε* = 0.8, respectively. At constant current density, *x*_1_ is linearly related to the maximum volume flux, *Q*_max_ = *v*_*c*_*x*_1_*W*, which is included as a secondary ordinate in Fig. 3(a). For small pore radii, we find that *x*_1_ ≈ *x*_1,drag_ ≪ *x*_1,stat_, indicating that the flow is primarily limited by the viscous resistance of the porous separator. In contrast, for *r* ≈ 50 µm, *x*_1_ becomes significantly smaller than *x*_1,drag_ and approaches *x*_1,stat_, demonstrating that gravity constitutes the dominant mechanism opposing the flow in this regime. The optimal pore radius, corresponding to the maximum attainable values of both *x*_1_ and *Q*_max_, is found in the transitional region between these two limiting regimes at approximately *r* ≈ 30 µm. However, it should also be noted that, in this case, the maximum electrode height is limited to less than 10 cm for this combination of current density and porosity.

**Fig. 3:**
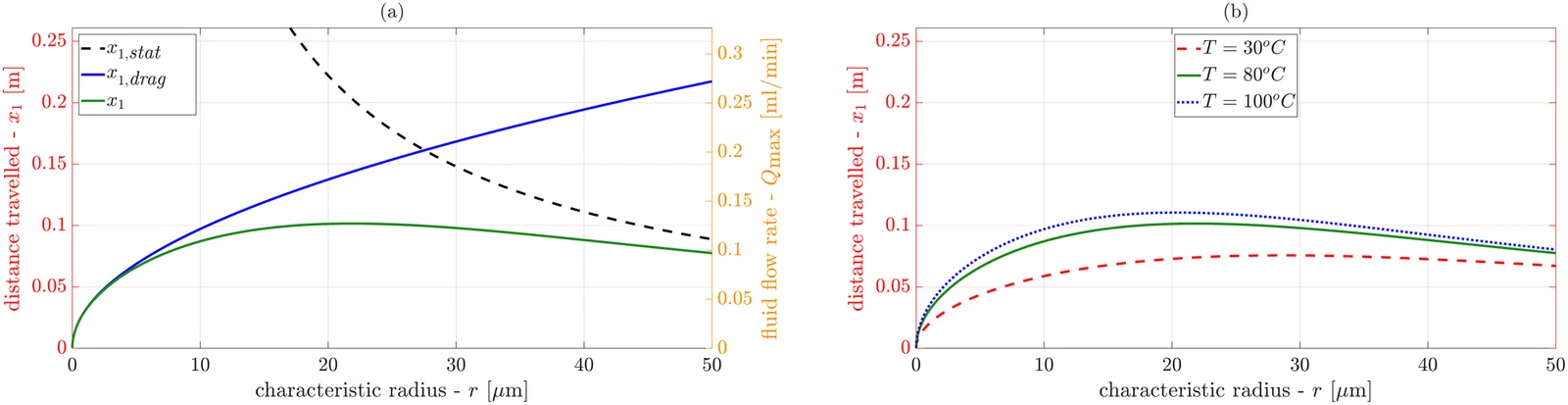
(a) Effect of the pore radius *r* on the flow in terms of distance travelled (*x*_1_, left axis) and flow rate (*Q*_max_, right axis), at *T* = 80 °C. Different effects are considered: drag (no gravity, *i.e.*, *g* = 0, blue line, eqn (22)), no flow (*i.e.*, no drag, *Q* = 0, with gravity *g* ≠ 0, dashed line, eqn (21)) and both flow and gravity (green line, eqn (23)). Results computed using *α*_*i*_ = *α*_2_. The porosity is *ε* = 0.80, and the current density *i* = 1 A cm^−2^ (corresponding to *v*_*c*_ = 1.0432 × 10^−6^ m s^−1^). (b) Effect of the temperature *T* on the distance travelled, *x*_1_, defined as in eqn (23). Temperature effects are considered in *µ*, *ρ* and *γ* following Hodges *et al.* (2023).^14^ The other flow properties are kept as in panel (a).

In order to evaluate the temperature dependence of these findings, *x*_1_ is plotted for parameters evaluated at three different temperatures in Fig. 3(b). It is observed that the achievable electrode height generally decreases with decreasing temperature, implying that the increase in viscosity at lower temperatures outweighs the corresponding increase in surface tension.

As shown in Fig. 4, where the current density *i* is varied at fixed *ε*, lower *i* leads to larger values of *x*_1_ (Fig. 4(a)). However, this increase is not sufficient to enhance the overall *Q*_max_, which is consistently higher for larger current densities, as shown in Fig. 4(b). The basic relation underlying this behaviour can be inferred from the case neglecting gravity (eqn (22)), for which 
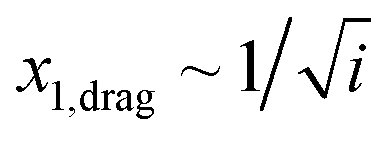
. Consequently 
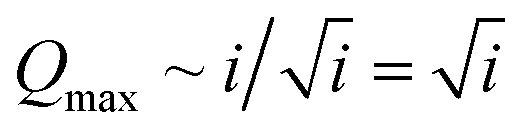
, implying that the overall production rate is always higher at larger currents for otherwise constant properties. Fig. 5 shows that decreasing the porosity drastically reduces both *x*_1_ and *Q*_max_ due to the increased flow resistance within the separator. As a consequence, viscous drag remains the dominant effect up to larger pore radii at lower values of *ε*. Finally, we note that the present configuration differs from the capillary flow model of Hodges *et al.*,^8^ who assumed a constant flux along the separator height to replicate their supplementary experiments and did not scale the electrode size with the separator height.

**Fig. 4:**
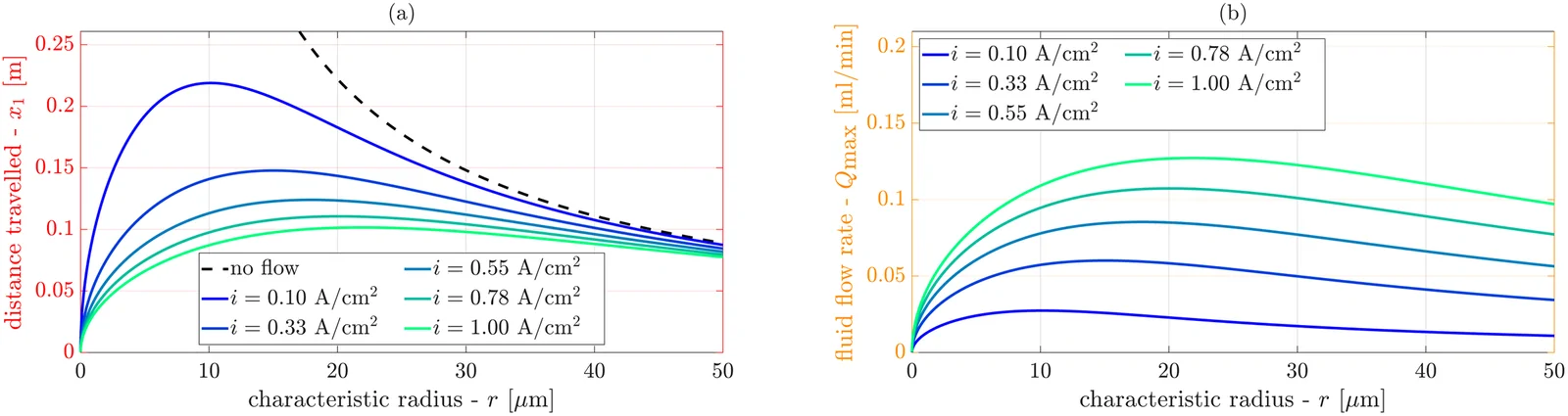
Variable current density and fixed porosity *ε* = 0.80. Effect of the pore radius *r* on the flow in terms of distance travelled (*x*_1_, panel a) and flow rate (*Q*_max_, panel b). Both flow and gravity are considered, and the solution corresponding to eqn (23) is reported. The dashed line in (a) corresponds to the no-flow condition (relative to the largest value of porosity); see eqn (21). Results computed using *α*_*i*_ = *α*_2_.

**Fig. 5:**
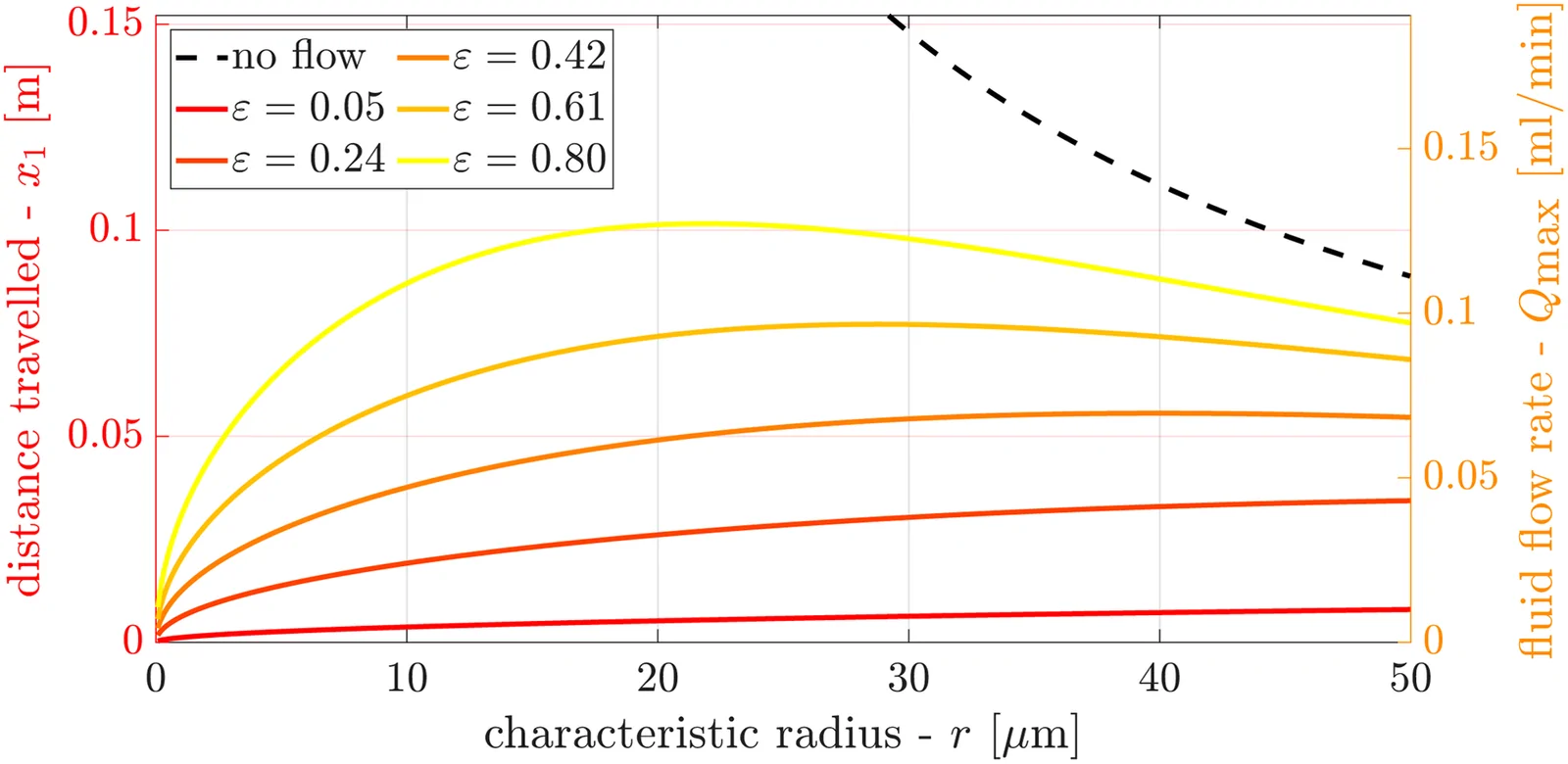
Fixed current density and variable porosity. Effect of the pore radius *r* on the flow in terms of distance travelled (*x*_1_, panel a) and flow rate (*Q*_max_, panel b). Both flow and gravity are considered, and the solution corresponding to eqn (23) is reported. The dashed line corresponds to the no-flow condition (relative to the largest value of porosity); see eqn (21). Results computed using *α*_*i*_ = *α*_2_ and *i* = 1.0 A cm^−2^, corresponding to *v*_*c*_ = 1.0432 × 10^−6^ m s^−1^.

#### 3D numerical simulations

Having established that, within the 1D model, the capillary electrolyzer concept cannot operate without reaching solubility limitations/crystallization, we explore the operation in three dimensions through the use of numerical simulations. Specifically, we simulate the geometry described in Hodges *et al.* (2022) for which continuous operation has been demonstrated to explore the case of smaller electrodes mounted on a larger-surface-area separator, which can also act as a reservoir and possibly enable a recirculation flow. In this way, we can further examine the mechanism through which this system could operate for long periods up to several days without substantial salt deposition/crystallization being observed experimentally.^8^

Recirculating flow in the present system may be driven by concentration-induced density gradients (buoyancy) or surface tension gradients (solutal Marangoni effects). In Fig. 6, the weight fraction of KOH at 0.1 A cm^−2^ is shown for the cathode side after a time of 18 000 s (5 hours). We first consider the case for which both Marangoni flow and buoyancy are absent. The corresponding results are shown in Fig. 6(a). Unlike in the 1D model, the maximum concentration is below the maximum solubility of KOH in water (∼60% at 80 °C). However, we are already beginning to approach the solubility limit even at the present current density of 0.1 A cm^−2^. This contrasts with the experimental results of Hodges *et al.*,^8^ where much higher current densities were sustained for a much longer time frame (0.3–0.4 A cm^−2^ for 30 days).

**Fig. 6:**
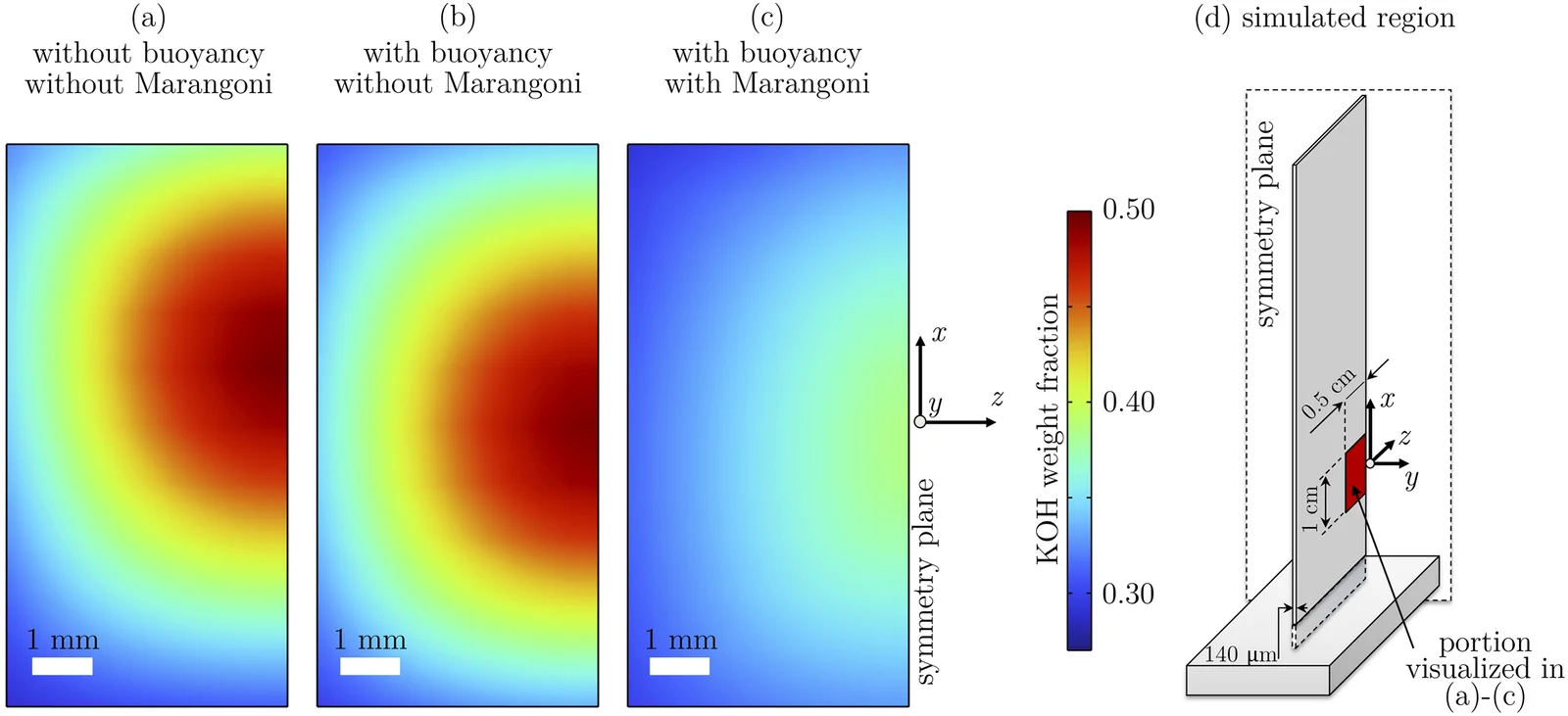
(a) KOH weight fraction on the electrode without buoyancy and Marangoni flows at 0.1 A cm^−2^ after 5 hours. (b) KOH weight fraction on the electrode with buoyancy and without Marangoni flows at 0.1 A cm^−2^ after 5 hours. (c) KOH weight fraction on the electrode with buoyancy and Marangoni flows at 0.1 A cm^−2^ after 5 hours. (d) Schematic indicating the position and dimensions of the region shown in panels (a)–(c).

The situation changes only slightly when including the buoyancy term, as shown in Fig. 6(b). Although buoyancy influences the overall behaviour of the system, its effect is primarily limited to a vertical shift of the profile due to the increased density of the KOH-rich fluid. The profile shape remained largely unchanged compared with the case without buoyancy in Fig. 6(a). There does not appear to be a substantial buoyancy-related recirculation. The lower steady-state concentrations can therefore be attributed primarily to enhanced diffusion, which, unlike in the 1D model, is able to redistribute species in all spatial directions.

The limited effect of buoyancy can be understood by considering the solutal Darcy–Rayleigh number 
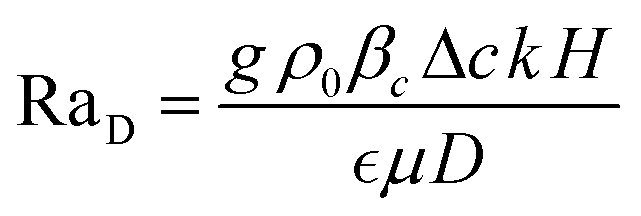
, where *β*_*c*_ is the solutal expansion coefficient, *k* is the permeability of the separator and *H* denotes a characteristic length scale. This dimensionless number quantifies the relative importance of buoyancy-driven flow and diffusive transport in a fluid-saturated porous medium. Assuming the upper limit Δ*ρ* = *ρ*_0_*β*_*c*_(*c*_max_ − *c*_0_) ≈ 440 kg m^−3^, we obtain, on the electrode scale (*H* = 1 cm), Ra_D_ = 179 or Ra_D_ = 28 when the permeability *k* is estimated using *α*_1,2_ (characteristic radius *r* = 8 µm), respectively. These values of Ra_D_ remain close to or below the onset of convection in porous media, occurring^31–33^ for the critical value Ra_D_ = 4π^2^ ≈ 40. Since Ra_D_ scales linearly with *H*, this calculation suggest that convection may become a relevant transport mechanism for larger electrodes.

Given the importance of diffusion established for the case without Marangoni convection, it is remarkable that successful operation has also been demonstrated at lower temperatures down to room temperature for either alkaline or acidic water electrolysis capillary electrolyzer systems.^34,35^ At such temperatures, the numerical result of achieving steady-state concentrations below the solubility limit is not expected to hold as the diffusivity of KOH and water would be markedly lower, which would impact the concentration profile in a manner that would favour crystallization in the absence of an additional convection-related term.

A potential driver for such convection is Marangoni flow, which has been shown to be of high importance for bubble coalescence/departure in electrolyzer systems previously. Given the apparent inability of a buoyancy-related instability to explain experimentally observed results, we also considered this in our simulation framework.^36–38^ In this case, we specifically examine the solutal Marangoni effect due to concentration gradients that can evolve on the electrode surface (as a porous gas-diffusion electrode with a high porosity of *ε* = 0.8, there will be a liquid–vapour interface) and as a thin wetting film layer on the separator itself. In simulations, this was performed using a slip condition – meaning that the hypothetical liquid film itself is infinitely thin but presumed contiguous – the validity of this will be discussed later.

As can be seen from Fig. 6(c), inclusion of a solutal Marangoni (in addition to buoyancy) effect led to a marked decrease in the maximum observed concentration at 0.1 A cm^−2^ for the same simulation time (5 hours). At the same time, for the cases of 0.3 and 0.4 A cm^−2^, which were experimentally operated for a period of 30 days, we found that inclusion of a solutal Marangoni effect also avoided the local concentration of KOH exceeding the solubility limit within the separator (Fig. 7). This was not the case when not including a Marangoni contribution; simulations with only a buoyancy effect still exceeded the solubility limit (within approximately 1 hour of simulation time).

**Fig. 7:**
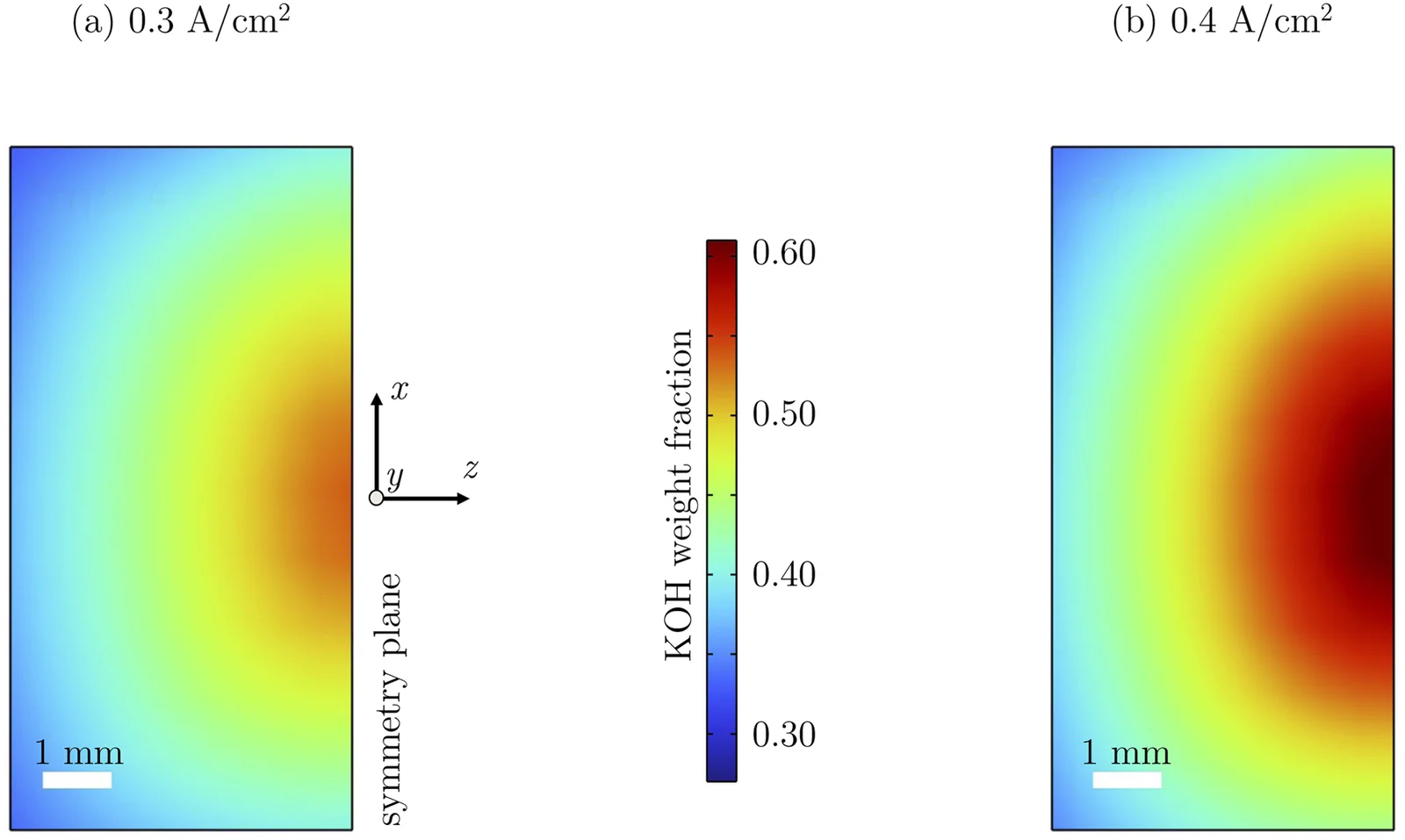
(a) KOH weight fraction on the electrode with Marangoni flows at 0.3 A cm^−2^ after 5 hours. (b) KOH weight fraction on the electrode with Marangoni flows at 0.4 A cm^−2^ after 5 hours. See Fig. 6(d) for the indication of the position and dimensions of the region shown in panels (a) and (b).

The evolution of the average concentration on the electrode surface with time is shown for various current densities in Fig. 8. It can be seen that, at 0.1 A cm^−2^, the concentration approaches a plateau regime at a KOH weight fraction of 0.34, although it continues to increase slightly even at the end of the 5 h simulation period. Similar trends are observed at the higher current densities of 0.3 A cm^−2^ and 0.4 A cm^−2^, which exhibit progressively higher plateau values and slightly more pronounced transients towards the end of the simulated 5 h period.

**Fig. 8:**
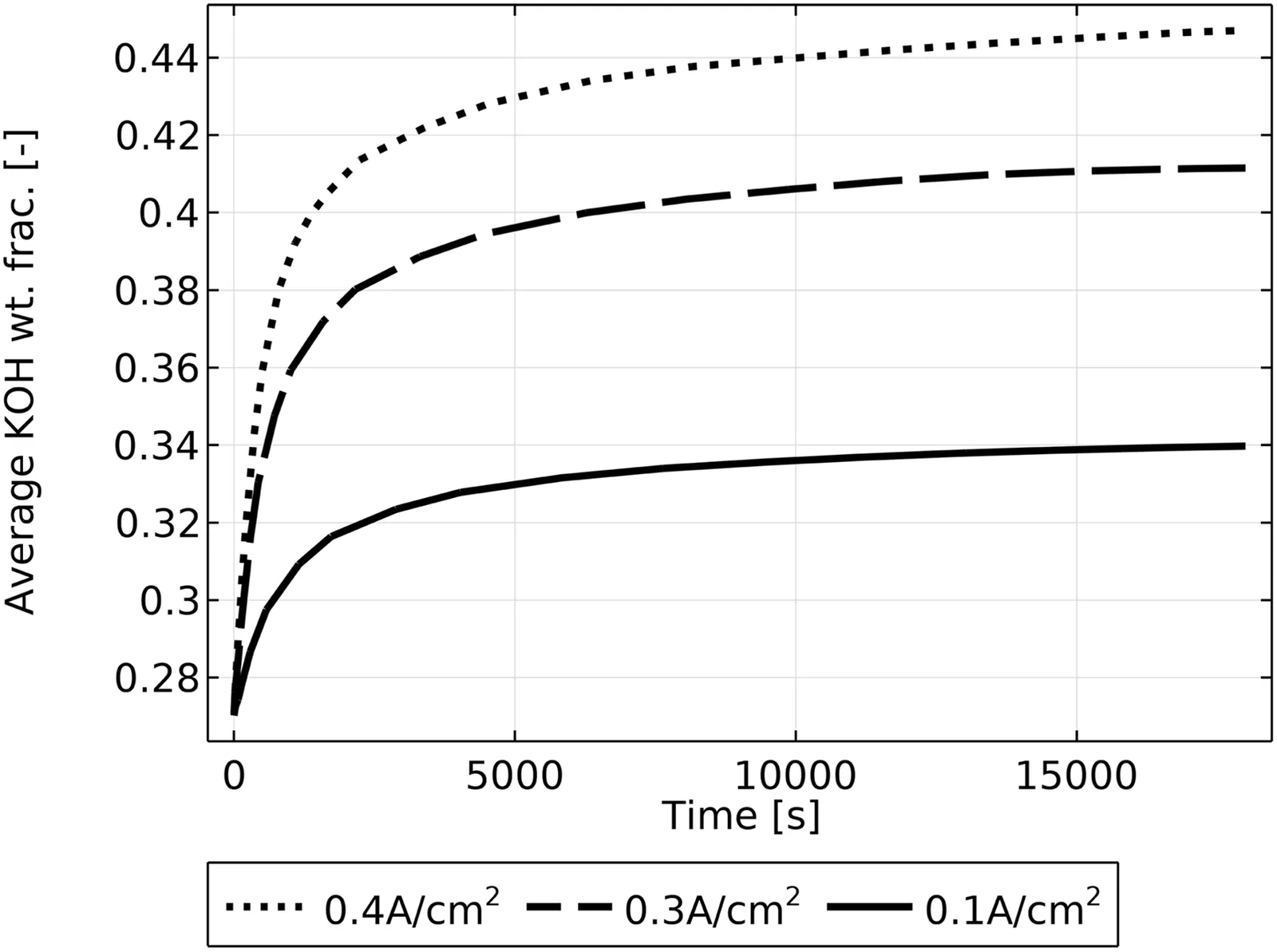
Average KOH weight fraction on the electrode *vs.* time at various current densities at 80 °C and with an initial concentration of 27 wt% for simulations including Marangoni flow.

In KOH solutions, the surface tension increment is positive, *i.e.*, *γ* increases with increasing electrolyte concentration. The resulting Marangoni flow at the surface is therefore ‘inward’ at the surface, resupplying electrolyte to the electrode area (high KOH concentration) from the surrounding regions at lower concentrations. This effect appears to dominate – the maximum velocity magnitude along the surface of the separator on the electrode was found to be slightly less than 1 mm s^−1^ for 0.1 A m^−2^ and somewhat greater than 1 mm s^−1^ at higher current densities (∼1.5 mm s^−1^ for both 0.3 and 0.4 A cm^−2^). This can be compared to an estimate of the Marangoni velocity 
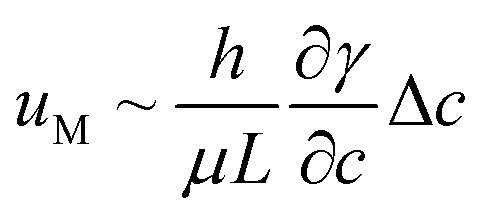
, which is obtained by balancing the Marangoni shear stress 
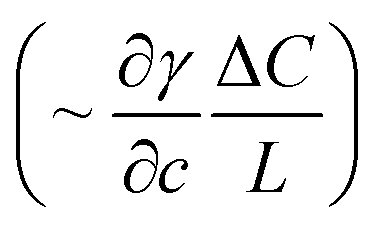
 with viscous shear in a liquid film of thickness *h*
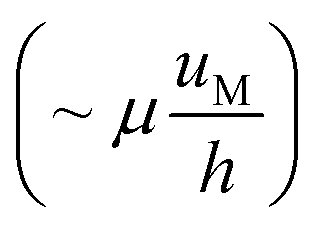
. Using the electrode size of 1 cm as the characteristic length scale *L* and adopting the KOH concentrations estimated on the electrodes, consistent values for *u*_M_ are obtained when assuming a film thickness *h* of approximately 1 µm, which is not unreasonable.

Importantly, the flow towards the electrode along the separator surface is balanced by a return flow away from the electrode in the centre plane of the separator, effectively reducing the concentration in the electrode area.

While the results indicate that Marangoni effects positively affect the range of continuous operation, there is still an upper limit to the current density that could be practically applied, even when considering Marangoni flows in this system. For the capillary electrolyzer experiments described in Hodges *et al.* (2022),^8^ current densities up to 1 A cm^−2^ were tested, albeit for much shorter time periods. At such high current densities, our simulation results indicate that, within approximately 10 minutes, the local KOH concentration would exceed the solubility limit at locations within the separator.

#### Underlying assumptions in the 3D simulations

The simulation results point towards a solutal Marangoni flow as possibly enabling the experimentally observed capillary electrolyzer behaviour, but there were a number of assumptions made to obtain these simulation results that deserve deeper discussion.

Firstly, in the absence of Maxwell–Stefan diffusion coefficients at high temperatures for KOH-water solutions, a simplified Fickian approach was taken (with equal diffusivities for KOH and water). Even this approach required some extrapolation. Conductivity data at 80 °C are available, but the validity of the Nernst–Einstein relationship for high concentrations means that converting these data to a salt diffusion coefficient (and assuming that the diffusivity of water in this mixture was equal) was questionable. In the interest of simplicity, the diffusivity data reported by Bhatia *et al.* (1968)^18^ was extrapolated using a 2nd-order polynomial approach with respect to temperature and concentration (the maximum temperature used in that work was 65 °C). In practice, this meant that the diffusion coefficients for KOH and water ranged between 7.5 × 10^−9^ and 9 × 10^−9^ m^2^ s^−1^ at the temperature we simulated for the range of physically plausible KOH weight fractions. This is, in principle, much higher than the values expected *via* estimation using the Nernst–Einstein, but when including Marangoni contributions, the overall impact of diffusion was minimal, particularly at higher current densities. This is to be expected as the problem shifts more and more towards a convection-dominated regime.

Other important physical properties, such as viscosity, surface tension, and density, were also taken from the literature (found in the comprehensive paper by Hodges *et al.* (2023)^14^), but due to the small deviations in all of these expressions, the overall uncertainty in the results shown here may be larger. The existence of a persistent thin-wetting film over the surface of the separator and the nature of the vapour–liquid (solid) interface at the gas diffusion electrode was simplified due to a lack of specific information.

Finally, we assume isothermal operation, but based on the conditions experimentally observed by Hodges *et al.* (2022)^8^ in their work with the capillary electrolyzer, examination of heat effects relating to the difference between applied cell potential and thermoneutral voltage would also be interesting to explore as the cell voltage is quite low compared to conventional electrolyzer systems.^39,40^ Non-isothermal operation would impact the diffusivity, viscosity, surface tension and density profiles, meaning that it could have significant implications for buoyancy-related effects, water and salt transport within the separator, and could introduce a possible role for thermal Marangoni convection in the operation of a capillary electrolyzer, which could also impact operation.^36^ It would also be important to establish if there were any effects related to the evaporation of liquid within the separator, as there are scant details on the humidity of the surrounding environment during operation of these systems.

## Conclusions

A modeling study was conducted to investigate the transport of water and KOH in capillary-fed electrolysis, a process introduced by Hodges *et al.* (2022).^8^ A cell with a porous separator of 140 µm was analyzed for current densities varying between 0.1 and 1.0 A cm^−2^ and an electrolyte of 27 wt% KOH at 80 °C. Two models were developed: a 1D analytical model and a 3D numerical model.

The 1D model was formulated and analytically solved for transport restricted to diffusion and capillary convection, after diffusio-osmotic effects were estimated to be negligible in the present system. The 1D model predicted that the maximum attainable electrode height, neglecting porosity effects on diffusion, was only 0.4 cm at 0.1 A cm^−2^ and 0.14 cm at 1.0 A cm^−2^. This maximum attainable electrode height was governed by a solubility limit, above which crystallization of KOH is expected to occur. Additional limitations imposed by the capillary feeding were found to be less stringent, allowing for electrode heights up to 10 cm at 1.0 A cm^−2^, with a non-monotonic dependence on the pore size. From this, we conclude that the sustained operation reported by Hodges *et al.* (2022)^8^ for an electrode height of 1.0 cm may not be explained by diffusion and capillary driving alone.

To investigate whether buoyancy or Marangoni flow can prevent excessive accumulation of KOH in the porous separator, 3D numerical simulations were performed for an electrode height of 1 cm at various current densities. A uniform temperature was assumed, meaning that both buoyancy and Marangoni flow were caused by local variations in the KOH concentration. It was shown that enhanced diffusion in all spatial directions helped to enable continuous operation, but the maximum KOH fraction at the electrode went up to 0.50 already after 5 hours running time at only 0.1 A cm^−2^, while the solubility limit was exceeded at 0.3 A cm^−2^. This situation did not change significantly when the effect of buoyancy was also considered.

However, the inclusion of Marangoni flow (in addition to buoyancy) appeared to be effective in further limiting the maximum KOH fraction at the electrode, resulting in maximum KOH fractions of approximately 0.40 and 0.52 at current densities of 0.1 A cm^−2^ and 0.3 A cm^−2^, respectively, but at 1.0 A cm^−2^, the solubility limit was exceeded within 10 minutes. Buoyancy and Marangoni convection due to thermal effects were not considered, but might be important, especially when temperature variations in the separator are large (greater than 10 °C).

In summary, while the precise mechanisms underlying continuous operation of capillary-fed electrolyzers remain unclear, the present results highlight the potential importance of Marangoni effects, which warrant further investigation.

Our findings also have implications for the implementation of capillary feeding in microgravity. In the absence of gravity, the gravity-limited regime vanishes, leaving *x*_1,drag_ as the governing limit, which increases monotonically with pore radius *r*. At the same time, larger pores would enhance Marangoni-driven recirculation, further improving mass transport. Together, these effects suggest that capillary-fed electrolyzers may operate over a substantially wider parameter range in space applications.

## Author contributions

M. De Paoli: writing – original draft, methodology, funding acquisition. J. A. Wood: writing – original draft, methodology, conceptualization A. W. Vreman: writing – review & editing, methodology. D. Krug: writing – original draft, writing – review & editing, conceptualization.

## Conflicts of interest

There are no conflicts to declare.

## Supplementary Material

FD-OLF-D6FD00128A-s001

## Data Availability

The 3D computational simulations were performed using COMSOL Multiphysics 6.4 and will be made available based on reasonable request. The 1D model data for this article, including the scripts to generate Fig. 2–5, are available at Zenodo at https://doi.org/10.5281/zenodo.22939567. Supplementary information (SI) is available. See DOI: https://doi.org/10.1039/d6fd00128a.

## References

[cit1] Lohse D. (2018). Phys. Rev. Fluids.

[cit2] Zhao X., Ren H., Luo L. (2019). Langmuir.

[cit3] Swiegers G. F., Terrett R. N. L., Tsekouras G., Tsuzuki T., Pace R. J., Stranger R. (2021). Sustainable Energy Fuels.

[cit4] Akay Ö., Bashkatov A., Coy E., Eckert K., Einarsrud K. E., Friedrich A., Kimmel B., Loos S., Mutschke G., Röntzsch L., Symes M. D., Yang X., Brinkert K. (2022). npj Microgravity.

[cit5] Yang Y., Li J., Yang Y., Lan L., Liu R., Fu Q., Zhang L., Liao Q., Zhu X. (2022). Appl. Energy.

[cit6] Rox H., Ränke F., Mädler J., Marzec M. M., Sokołowski K., Baumann R., Hamedi H., Yang X., Mutschke G., Urbas L., Lasagni A. F., Eckert K. (2025). ACS Appl. Mater. Interfaces.

[cit7] Bashkatov A., Park S., Demirkır Ç., Wood J. A., Koper M. T. M., Lohse D., Krug D. (2024). J. Am. Chem. Soc..

[cit8] Hodges A., Hoang A. L., Tsekouras G., Wagner K., Lee C.-Y., Swiegers G. F., Wallace G. G. (2022). Nat. Commun..

[cit9] Tiwari P., Tsekouras G., Swiegers G. F., Wallace G. G. (2018). ACS Appl. Mater. Interfaces.

[cit10] Tiwari P., Tsekouras G., Wagner K., Swiegers G. F., Wallace G. G. (2019). Int. J. Hydrogen Energy.

[cit11] Tsekouras G., Terrett R., Yu Z., Cheng Z., Swiegers G. F., Tsuzuki T., Stranger R., Pace R. J. (2021). Sustainable Energy Fuels.

[cit12] Li J., Zhu Y., Chen W., Lu Z., Xu J., Pei A., Peng Y., Zheng X., Zhang Z., Chu S., Cui Y. (2019). Joule.

[cit13] Likhachev E. (2003). Tech. Phys..

[cit14] Hodges A., Renz S., Lohmann-Richters F., Al-Musawi A., Jupke A., Lehnert W., Swiegers G. F., Wallace G. G. (2023). J. Chem. Eng. Data.

[cit15] Schalenbach M., Tjarks G., Carmo M., Lueke W., Mueller M., Stolten D. (2016). J. Electrochem. Soc..

[cit16] Chung D.-W., Ebner M., Ely D. R., Wood V., Edwin García R. (2013). Modell. Simul. Mater. Sci. Eng..

[cit17] Tjaden B., Cooper S. J., Brett D. J., Kramer D., Shearing P. R. (2016). Curr. Opin. Chem. Eng..

[cit18] Bhatia R., Gubbins K., Walker R. (1968). Trans. Faraday Soc..

[cit19] Vogel W. M., Routsis K. J., Kehrer J., Victor J., Landsman D. A., Tschinkel J. G. (1967). J. Chem. Eng. Data.

[cit20] Schindler M., Ajdari A. (2009). Eur. Phys. J. E:Soft Matter Biol. Phys..

[cit21] Bacchin P., Leng J., Salmon J.-B. (2022). Chem. Rev..

[cit22] Akdeniz B. , PhD Thesis - Research UT, Graduation UT, University of Twente, Netherlands, 2024

[cit23] Marbach S., Bocquet L. (2019). Chem. Soc. Rev..

[cit24] Gupta A., Shim S., Stone H. A. (2020). Soft Matter.

[cit25] Coday B. D., Luxbacher T., Childress A. E., Almaraz N., Xu P., Cath T. Y. (2015). J. Membr. Sci..

[cit26] Meulenbroek A., Deen N., Vreman A. (2024). Electrochim. Acta.

[cit27] Ellison W. J., Lamkaouchi K., Moreau J.-M. (1996). J. Mol. Liq..

[cit28] Susanto H., Ulbricht M. (2009). J. Membr. Sci..

[cit29] Meng X. (2017). et al.. Front. Environ. Sci. Eng..

[cit30] Nield D. A. , Bejan A. et al., Convection in Porous Media, Springer, 2006, vol. 3

[cit31] Horton C., Rogers Jr F. (1945). J. Appl. Phys..

[cit32] Lapwood E. (1948). Math. Proc. Cambridge Philos. Soc..

[cit33] De Paoli M. (2023). Eur. Phys. J. E.

[cit34] Hoang A. L., Owen R. E., Tsekouras G., Brett D. J. L., Swiegers G. F. (2023). Sustainable Energy Fuels.

[cit35] Tripathi S., Subbaiah R., Rajendran M., Vengatesan S., Subramanyan V. (2026). ChemCatChem.

[cit36] Park S., Lohse D., Krug D., Koper M. T. (2024). Electrochim. Acta.

[cit37] Babich A., Bashkatov A., Eftekhari M., Yang X., Strasser P., Mutschke G., Eckert K. (2025). PRX Energy.

[cit38] Park S., Liu L., Demirkır Ç., van der Heijden O., Lohse D., Krug D., Koper M. T. (2023). Nat. Chem..

[cit39] LeRoy R. L., Bowen C. T., LeRoy D. J. (1980). J. Electrochem. Soc..

[cit40] LeRoy R. L. (1983). J. Electrochem. Soc..

