# Supplementary material for: Modelling and simulation of species transport in capillary-fed electrolysis cells
Source: Faraday Discuss. 2026 Oct 27. Online ahead of print. doi: 10.1039/d6fd00128a (PMC13757278; doi:10.1039/d6fd00128a)
Supplement: Supplementary file 1 [file FD-OLF-D6FD00128A-s001.pdf]

Supplementary Information (SI) for Faraday Discussions.
This journal is © The Royal Society of Chemistry

Modelling and simulation of species transport in capillary-fed electrolysis cells: Supplementary Information

Marco De Paoli,[a] Jeffery A. Wood,[b] Albertus W. Vreman[c,d] and Dominik Krug,*[e,f]

Received 00th January 20xx, Accepted 00th January 20xx DOI: 10.1039/x0xx00000x

Additional notes on the 1D model

Pressure drop within a porous material

Assuming the membrane as a homogenous and isotropic porous medium having porosity ε, the pressure gradient in longitudinal direction dp/dx can be expressed as proportional to the gap averaged flow velocity u following the Darcy law:[1]

$$u(x) = -\frac{k}{\mu(T)}\frac{dp}{dx}, \tag{S1}$$

where $\mu(T)$ is the fluid viscosity at temperature T (obtained from Hodges *et al.*[2]) and k is the permeability of the medium. The Kozeny-Carman formulation[3] can be employed to determine the permeability, which reads:

$$k = \frac{\varepsilon^2 r^2}{45(1-\varepsilon)^2}, \tag{S2}$$

where r is the characteristic pore radius. As a result, considering Eq. (3), we obtain that the coefficient α_1 to be employed in Eq. (16) is

$$\alpha_1 = \frac{W\delta}{\mu(T)}\frac{\varepsilon^2 r^2}{45(1-\varepsilon)^2}. \tag{S3}$$

Pressure drop within a pipe

An alternative approach proposed by Hodges *et al.*[4] consists of considering the membrane as an array of tubes, and computing the pressure gradient consequently. In particular, assuming the medium homogeneous, isotropic and with porosity ε, it can be modelled as an array of tubes, the effective length of which, at the coordinate x, is x/τ where $\tau = (2-\varepsilon)^2/\varepsilon$ is the tortuosity of the medium. The mean flow in each tube is proportional to the pressure gradient:

$$\hat{u}(x) = -\frac{r^2}{8\mu(T)\tau}\frac{dp}{dx}. \tag{S4}$$

Therefore, the mean velocity in x direction is $u(x) = \varepsilon\hat{u}$ corresponding to a flow rate:

$$Q(x) = u(x)W\delta = -\frac{r^2}{8\mu(T)}\frac{\varepsilon^2 W\delta}{(2-\varepsilon)^2}\frac{dp}{dx}. \tag{S5}$$

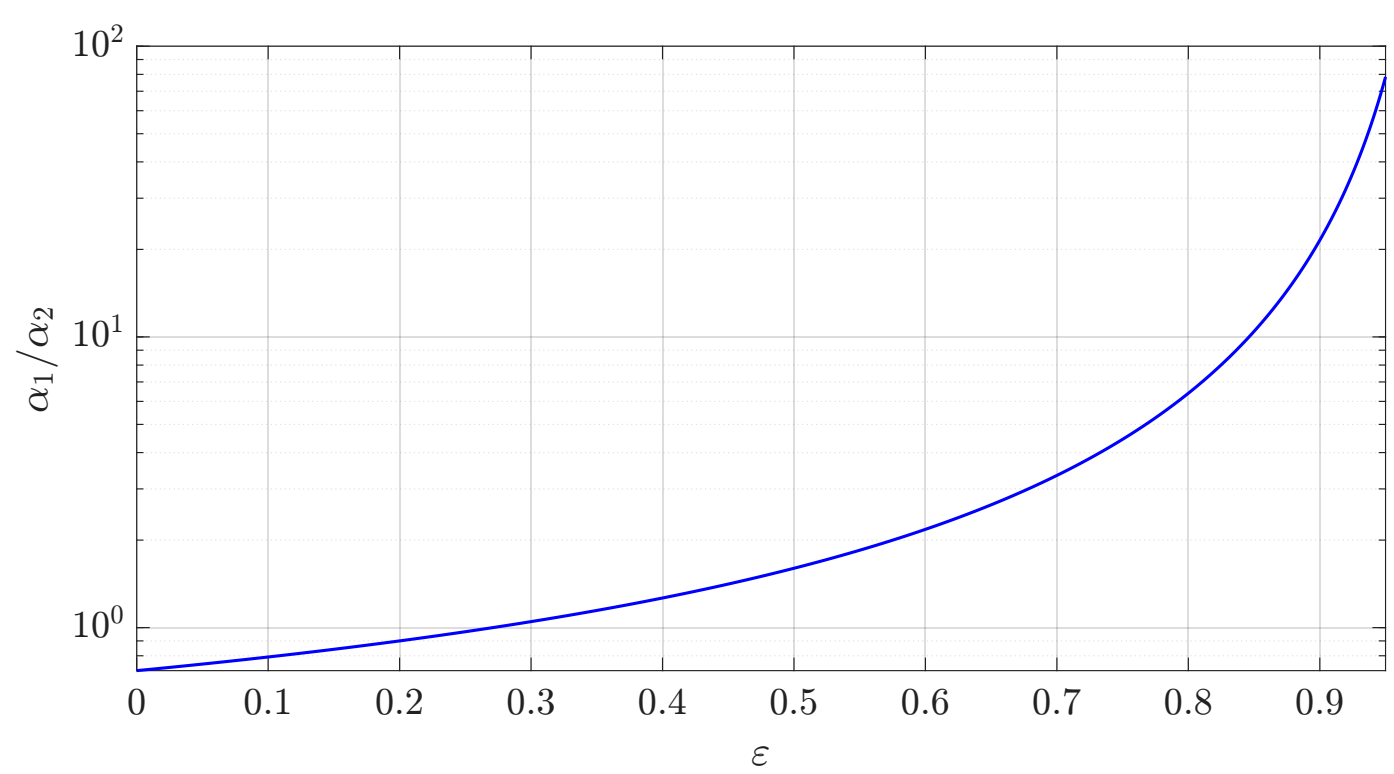

Figure S1 Ratio of the coefficients α_1 and α_2 obtained in Eq. (S3) and Eq. (S6), respectively, as a function of the porosity ε.

[a] *Institute of Fluid Mechanics and Heat Transfer, TU Wien, Getreidemarkt 9, Wien 1060, Austria*
[b] *Soft Matter, Fluidics and Interfaces, MESA+ Institute for Nanotechnology, J. M. Burgers Centre for Fluid Dynamics, University of Twente, Enschede 7500 AE, Netherlands*
[c] *Nobian Innovation Center, Hengelosestraat 500, 7522 NH Enschede, The Netherlands*
[d] *Power and Flow group, Department of Mechanical Engineering, Eindhoven University of Technology, PO Box 513, 5600 MB, Eindhoven, The Netherlands*
[e] *Chair of Fluid Mechanics and Institute of Aerodynamics, RWTH Aachen University, Aachen 52062, Germany*
[f] *Physics of Fluids Group, Max Planck Center for Complex Fluid Dynamics and J. M. Burgers Centre for Fluid Dynamics, University of Twente, Enschede 7500 AE, Netherlands. E-mail: d.krug@aia.rwth-aachen.de*

As a result, the coefficient α_2 to be employed in Eq. (16) is:

$$\alpha_2 = \frac{W\delta}{\mu(T)} \frac{\varepsilon^2 r^2}{8(2-\varepsilon)^2}. \tag{S6}$$

The Kozeny–Carman form (S2), $k \propto (1-\varepsilon)^{-2}$, treats the medium as a packed bed of solid grains, with drag scaling from the wetted surface area of discrete particles; it recovers $k \to 0$ as $\varepsilon \to 0$ and $k \to \infty$ as $\varepsilon \to 1$. The alternative form (S6), $k \propto (2-\varepsilon)^{-2}$, instead models the medium as a bundle of tortuous capillary channels, with the tortuosity closure $\tau = (2-\varepsilon)^2/\varepsilon$ reducing to $\tau = 1$ (straight, unobstructed capillaries) as $\varepsilon \to 1$. The $(2-\varepsilon)^{-2}$ formulation is more appropriate for membranes, which are typically better described as a network of interconnected pores threading through a continuous solid matrix rather than as a bed of discrete particles, so the tortuous-tube-bundle picture (and its correct $\varepsilon \to 1$ limiting behaviour) matches the membrane geometry more closely. Therefore, the expression (S6) is employed to model the system in this case.

The two formulations are not distinctly different in practice: over an intermediate porosity range (roughly $\varepsilon \approx 0.3-0.7$) the ratio α_1/α_2 stays within about an order of magnitude of unity, so the two models give broadly comparable permeabilities there; the divergence becomes significant only as $\varepsilon \to 0$ or $\varepsilon \to 1$, where the underlying geometric assumptions differ most (Fig. S1).

Additional 3D simulation framework details

The physical properties for concentrated KOH were taken primarily from correlations found in Hodges *et al.* (2023) as well as the diffusivity data of Bhatia *et al.* (1968). For density, the correlation found in Hodges *et al.* (2023) was used, the viscosity was calculated using the expression found within with the pure water viscosity term determined using the correlation by Likhachev et al. also found within that reference. Surface tension was determined using the Ripoche-Rolin expression in that work as well, this does mean there is extrapolation beyond the range of 50wt%.

The partial differential equations solved relate to the momentum and continuity (velocity components, pressure and density) and to the transport of KOH & water, described by

$$\rho \frac{\partial w_i}{\partial t} + \nabla \cdot \vec{j}_i + \rho \vec{u} \nabla w_i = 0, \tag{S7}$$

$$\vec{j}_i = -\rho D_i \nabla w_i, \tag{S8}$$

where w_i is the weight fraction of component i, ρ is the mixture density, D_i the diffusivity of component i and $\vec{u}$ the velocity vector. Since the weight fractions of KOH and water are not independent, only one was solved for. At the reservoir inlets (outer walls of the reservoir), the weight fraction of KOH was set to 0.27 (constant Dirichlet) and at the cathode and anode a respective out or influx of water set and a zero flux for KOH (electrode walls were impermeable to KOH). All other boundaries were zero flux for both KOH and water (which is also a symmetry condition).

As discussed in the main text, the porous media was taken in a Darcian fashion with a Kozeny-Carman relationship for permeability vs. porosity and a Bruggeman relationship for diffusivity in the porous media. The flow was assumed to be creeping (Stokes flow) in the fluid reservoir. A slip condition + weak and pointwise constraints were used to enforce Marangoni stresses related to surface tension gradients (+ in/outflow on the relevant electrode walls), a constant pressure condition on the reservoir inlets (outer walls of reservoir) and no-slip otherwise.

Notes and references

[1] D. A. Nield, A. Bejan *et al.*, *Convection in porous media*, Springer, 2006, vol. 3.

[2] A. Hodges, S. Renz, F. Lohmann-Richters, A. Al-Musawi, A. Jupke, W. Lehnert, G. F. Swiegers and G. G. Wallace, *Journal of Chemical & Engineering Data*, 2023, **68**, 1485–1506.

[3] J. Bear, *Dynamics of fluids in porous media*, Courier Corporation, 2013.

[4] A. Hodges, A. L. Hoang, G. Tsekouras, K. Wagner, C.-Y. Lee, G. F. Swiegers and G. G. Wallace, *Nature communications*, 2022, **13**, 1304.

[5] R. Bhatia, K. Gubbins and R. Walker, *Transactions of the Faraday Society*, 1968, **64**, 2091–2099.
